# Differences and common ground in the frameworks of health-related quality of life in traditional Chinese medicine and modern medicine: a systematic review

**DOI:** 10.1007/s11136-024-03669-1

**Published:** 2024-05-13

**Authors:** Yifan Ding, Zhuxin Mao, Nan Luo, Zhihao Yang, Jan Busschbach

**Affiliations:** 1 Department of Psychiatry, Erasmus MC, University Medical Center Rotterdam, Rotterdam, The Netherlands; 2 Centre for Health Economics Research and Modelling Infectious Diseases, Vaccine and Infectious Disease Institute, University of Antwerp, Antwerp, Belgium; 3 Saw Swee Hock School of Public Health, National University of Singapore, Singapore, Singapore; 4 Health Services Management Department, Guizhou Medical University, Guiyang, China

**Keywords:** Framework, Health-related quality of life, Chinese, Traditional Chinese medicine, Modern medicine

## Abstract

**Purpose:**

This systematic review aims to explore the conceptualization of health-related quality of life (HRQoL) in China. With HRQoL influenced by both modern medicine (MM) and traditional Chinese medicine (TCM), the study seeks to identify differences and common ground between the frameworks of MM and TCM as defined in the literature.

**Method:**

A systematic literature search was conducted across three Chinese databases and four English databases. The data was extracted including title, author(s), publication year, region, aim, method, category, and result. When sorting data, we broke down the HRQoL frameworks into concepts, domains and facets, with a focus on overlapped facets between the frameworks of MM and TCM.

**Results:**

A total of 31 studies were included. In the perspective of TCM, HRQoL is centered around three key 'concepts': (1) 'xingshentongyi' (unity of body and spirit), (2) 'tianrenheyi' (harmony between man and nature), and (3) 'qiqing' (seven emotional forms). In contrast, the MM framework comprises 'physical,' 'mental,' 'social,' and 'environment' domains. Out of the 59 unique facets identified, 28 are common to both TCM and MM, 9 specific to TCM, and 22 specific to MM. 'Appetite,' 'sleep,' and 'energy' are the most frequently mentioned facets in both frameworks.

**Conclusion:**

The concept of HRQoL in China encompasses frameworks rooted in both TCM and MM. While TCM and MM have distinct healthcare approaches, they share overlapping domains when measuring HRQoL through questionnaires. Furthermore, TCM and MM demonstrate considerable convergence in terms of HRQoL facets, showing the potential for utilizing HRQoL instruments across different cultural settings.

**Supplementary Information:**

The online version contains supplementary material available at 10.1007/s11136-024-03669-1.

## Plain English summary

Health-related quality of life (HRQoL) is about how people perceive their own health. The instruments used to measure it, even in China, are mostly devised in the West. However, it is increasingly understood that cultural distinctions might impact how we understand HRQoL, causing doubts about whether Western tools accurately represent this concept. This suggests that instruments created in the West might not fully grasp the health experiences of people in China, considering their unique cultural background. This systematic review study tries to understand from the literature how Chinese people think about HRQoL. It found two different ways of thinking about it: one based on modern medicine and the other on traditional Chinese medicine. Surprisingly, these ways of thinking lead to similar ideas about HRQoL. This study demonstrates the adaptability of HRQoL instruments across cultural settings with minimal adjustments. This challenging the notion that diverse cultures yield entirely distinct perspectives on HRQoL.

## Introduction

Health-related quality of life (HRQoL) is a multi-dimensional concept. Despite the challenge of defining HRQoL, there is a broad consensus that HRQoL focuses on revealing people’s subjective evaluation of health [1–4]. Obtaining HRQoL information is crucial for comprehending patient health, making informed medical decisions [5, 6], evaluating healthcare interventions, guiding public health policies, and ultimately contributing to evidence-based medicine [7–9]. Some HRQoL outcomes are preference-based, informing decisions on health products, technologies, and policies through QALY calculations [7]. Note that in this paper, we focus on HRQoL, rather than quality of life (QoL) in general.

The way we conceptualize HRQoL determines the specific health facets that we include in HRQoL measurements. To ensure valid and reliable HRQoL observations, standardized instruments are utilized. However, the recognition of cultural influences on HRQoL conceptualization has led to discussions about the suitability of Western-developed HRQoL measures in China [10, 11]. Specifically, when Western instruments, grounded in a distinct cultural context, are applied to measure HRQoL in China, dimensions important to the Chinese population's understanding of health might be overlooked or not accurately captured. This misalignment can lead to an incomplete representation of the true HRQoL experienced by Chinese individuals [12].

For example, Prior et al. found that participants from Cantonese-speaking communities in England described HRQoL using facets related to traditional Chinese medicine (TCM), such as ‘demons’, ‘food’, and ‘weather’, alongside common Western facets [13]. Similarly, a Q-methodological study emphasized the importance of 'spirits' and 'body constitution' in describing HRQoL among Chinese living in China, which were often absent in commonly used instruments [11]. Additionally, Mao et al. compared Western and Chinese-developed HRQoL instruments and identified exclusive HRQoL domains in China, including ‘emotion control’, ‘weather adaption’, ‘social adaption’, ‘spirit’, and ‘complexion’ [8]. These new aspects of Chinese HRQoL appear to be linked to TCM, indicating that TCM could influence how people perceive and describe HRQoL. Alternatively, it's possible that the Chinese perspective on HRQoL shapes practices within TCM. Thus, it can be argued that Western-developed instruments may inadequately capture the health experiences of Chinese populations within their cultural context, for instance in neglecting the influence of TCM [8, 10, 14]. Consequently, using Western-developed HRQoL instruments to assess HRQoL in Chinese populations may not yield optimal results if there are conceptual differences between Western and Chinese perspectives [10, 12].

Despite debates regarding the applicability of Western HRQoL instruments, the influence of culture on HRQoL conceptualization and instrument development remains unclear [15]. In China, efforts have been made by the scientific community to define HRQoL within a Chinese cultural context and use it to guide instrument development. However, there has been no systematic assessment, comparison, or synthesis of these Chinese-specific definitions or instruments. Consequently, it is still unclear how HRQoL is defined in China, and how these ‘Chinese definitions’ differ from those used in imported HRQoL instruments. This study aims to systematically review published studies that describe the theoretical and operationalised conceptualization of HRQoL. The objective is to explore and synthesize perceptions of HRQoL within a Chinese cultural setting.

## Method

This systematic review followed the guideline of Preferred Reporting Items for Systematic Reviews and Meta-Analyses (PRISMA) [16], including the following four parts: search strategy, identification and selection, data extraction and quality assessment.

### Search strategy and inclusion criteria

A comprehensive search was conducted across three Chinese databases (CNKI, Weipu, and Wanfang) and four English databases (EMBASE, MEDLINE, Web of Science, and Cochrane), in addition to a restricted search on Google Scholar. We applied specific inclusion criteria: (a) reporting HRQoL measures developed in a Chinese cultural setting; (b) discussing the definition of HRQoL in Chinese cultural setting or constructing a conceptual framework of HRQoL specifically to Chinese culture; (c) qualitative interviews exploring Chinese people's understandings of HRQoL. We excluded articles focusing on Western-based perspectives of HRQoL.

Both of detailed Chinese and English search strategies were presented in the Appendix 1. The Chinese search strategy was developed through an internal discussion between the four Chinese researchers in team. When formulating the English strategy, we sought professional assistance from the librarians at Erasmus MC, followed by a discussion within the entire research team. To ensure a comprehensive search, we took into account potential spelling errors and synonyms when formulating the search strategies, as can be seen in the search strategies presented in the Appendix 1.

### Identification, selection and exclusion criteria

The research team reviewed all types of publications including original research, reviews, commentaries and dissertations. However, conference reports were excluded due to their potential lack of rigor and incomplete data. There were no constraints based on publication date other than the specified end date (March 3rd, 2022).

Two bilingual reviewers (YD and ZM) reviewed the titles and abstracts, applying the following exclusion criteria: (a) studies focused on a specific disease; (b) studies focused on a particular population (e.g., elderly or specific region); (c) studies utilizing existing instruments such as SF-36, EQ-5D, etc.; (d) studies focused on other constructs (e.g., happiness, well-being, life satisfaction); (e) studies focused on the target population which is not Chinese. Any disagreements between the reviewers were resolved through internal team discussions (ZM, ZY, and NL). The first author (YD) then conducted independent reviews of the resulting full-text articles. In cases where YD had doubts regarding the eligibility of a paper, the doubts were discussed with the review team. The review team consisted of one member with methodological expertise (ZM) and two individuals well-versed in the topic (ZY and NL). If disagreements persisted, they were resolved through consensus, but in case a consensus was not reached, an external opinion (JB) was sought.

### Data extraction and analysis

According to the searching results, the key information of all eligible studies was extracted including the title, author, publish year, region, study aim, methods, and results (see Appendix 2). After reviewing, all eligible articles were categorized into two groups based on their content: definition and framework. The definition category focused on providing a general definition of HRQoL, while the framework category presented a detailed hierarchical system organizing specific health facets into health domains, and further grouped into health concepts. The category of each paper is documented in Appendix 2. In the “Results” section, we extracted specific definitions from papers focusing on the general definition. For those paper that focused on the frameworks, we outlined the main hierarchical structure of domains and concepts. The specific facets for each instrument were documented in Appendix 3.

This review aimed to synthesize the conceptualization of HRQoL, both theoretically (narrative definition) and operationally (HRQoL instrument presentation). In our approach to HRQoL frameworks, we employed a four-level hierarchy, which included concepts (the first level), domains (the second level), facets (the third level), and items (the fourth level). 'Concepts' represent the higher-order theoretical components of the theory, while 'domains' are the second-order aspects defined using 'items.' Given that closely related items may have different wording (e.g., walking, mobility, movement), we grouped similar items under the term 'facets'. In this study, we looked for the overlap in facets between MM and TCM. However, during the analysis process, we observed that some studies presented HRQoL from a TCM perspective, while others did not. These two perspectives resulted in two distinct conceptual frameworks (see “Results” section, Figs. 3 and 4).

For studies focusing on a general definition of HRQoL, frequently-mentioned health concepts (sometimes also health domains) were extracted. For studies providing detailed descriptive system, all specific facets were extracted. The extracted facets (see Appendix 3) were grouped by the reviewers, and the frequency of each facet was recorded (see Appendix 4). During the grouping process, we referred to the existing categories of descriptive systems as found in papers because most of them were already grouped by the designers of frameworks or instruments. If facets were classified into different groups in different frameworks or instruments, we made the classification after discussions within the review team. The grouping was independently conducted by two reviewers (YD and ZM), and any inconsistencies were resolved through internal team discussions.

### Quality assessment

Normally, quality assessment for eligible studies is essential to a systematic review. However, after internal discussion, we opted not to conduct such an assessment in this review for maximizing the inclusion of studies. We were concerned that performing a quality assessment might inadvertently exclude relevant studies, resulting in the loss of valuable information. Nevertheless, the paper clearly written outside the scientific domain was excluded. These were articles that lacked 'scientific' jargon and failed to provide sufficient literature references to support the claims made in the article. For instance, it was found that a paper titled '20 New Concepts of Healthy Living,' which appeared more like a glossy magazine article than a scholarly contribution. As a result, such articles were considered unsuitable for inclusion in our study.

## Results

**Fig. 1:**
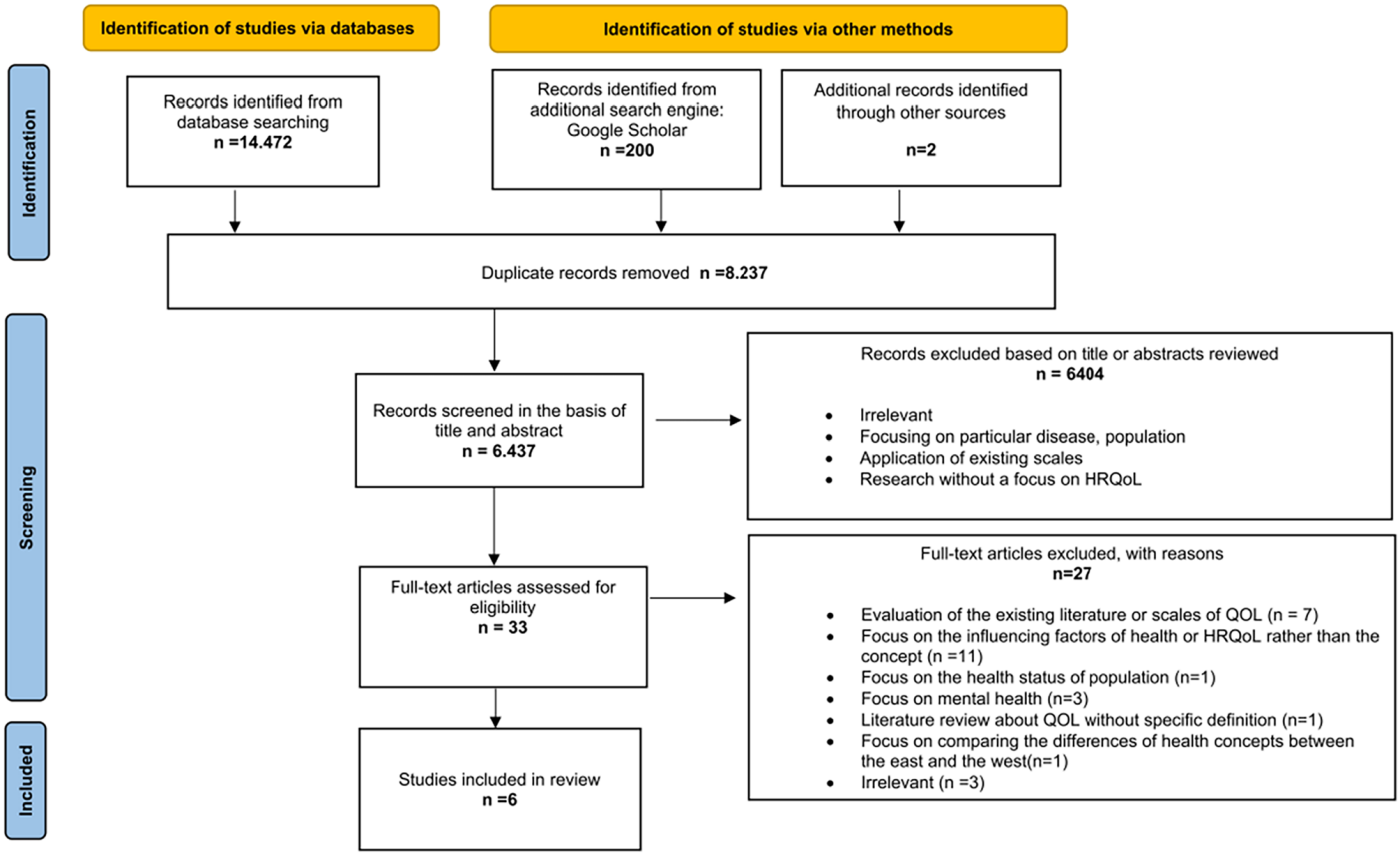
PRISMA flow diagram (English database)

**Fig. 2:**
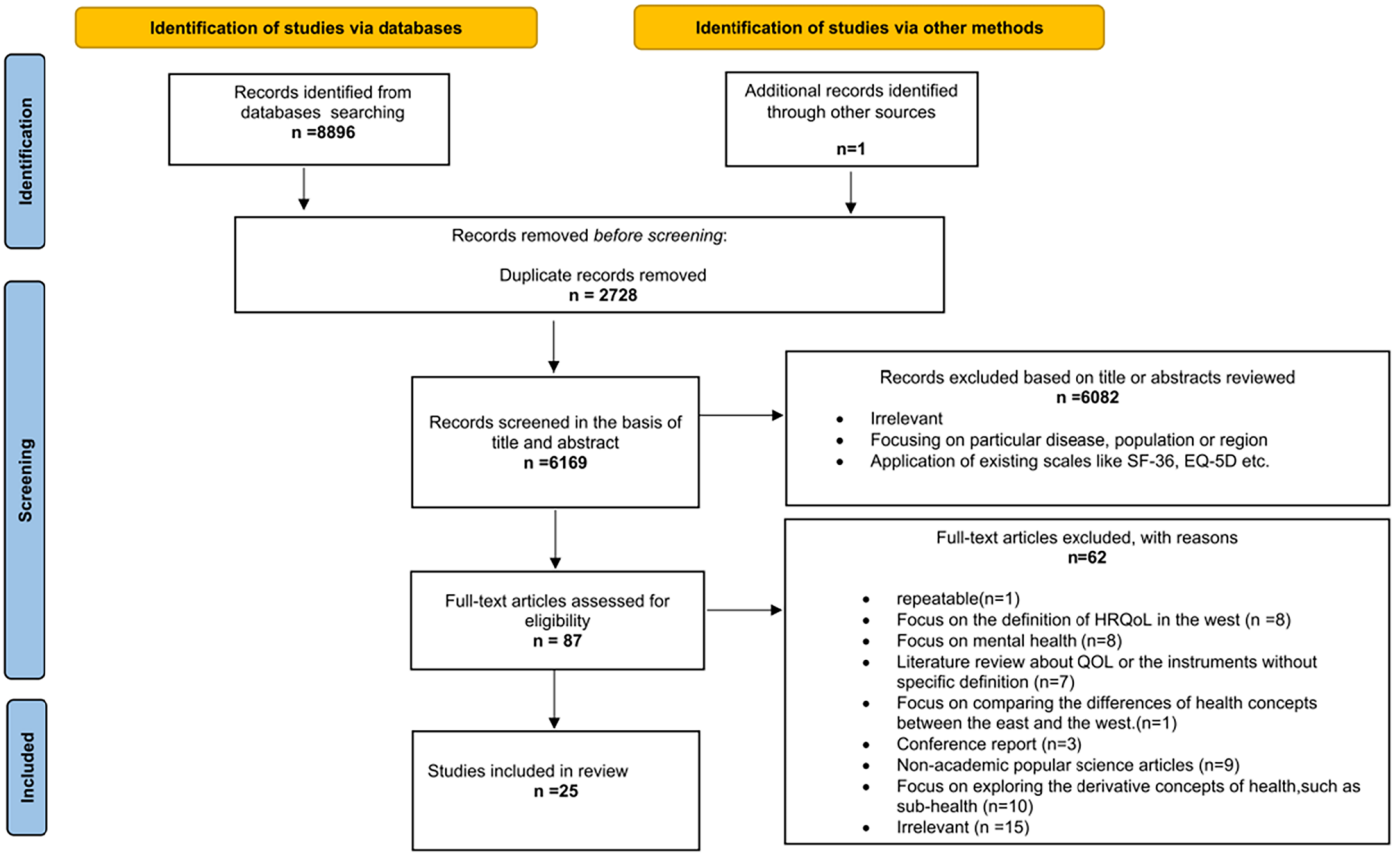
PRISMA flow diagram (Chinese database)

After removing duplicates, a total of 6437 English articles and 6169 Chinese articles were identified through database searches. Following title and abstract screening, 33 English studies and 87 Chinese studies remained. Full-text selection further reduced the number to 6 English articles and 25 Chinese articles. The detailed information of the included paper was provided in Appendix 2. The selection process was illustrated in Figs. 1 and 2 of the PRISMA flow charts.

During full-text review, the 31 included papers were primarily divided into two categories: focusing on general definitions of HRQoL and frameworks of HRQoL. Among them, 14 papers focus on providing a general definition of HRQoL, 11 papers delve into HRQoL frameworks, while 6 papers contain information pertaining to both the general definition and the framework. Additionally, a distinction was made between studies based on TCM perspectives and those using the perspective of modern medicine (MM). The categorization of each paper was provided in Appendix 2.

### The perspective of TCM on HRQoL

Based on the included papers focusing on general definition, we found that these papers introduced the foundational theory of TCM, which not only supplied potential corroborative evidence for TCM framework but also laid the foundation upon which TCM framework was developed.

The notion of 'Yin Yang' theory emerged most prominently as the foundational principle of TCM [17–26]. Additionally, the notion of 'Ping ren' [21, 22, 27–32] and 'He' [33, 34] were also highlighted as the basis principle of TCM. The meanings of these principles are as follows. According to TCM, everything in the universe can be divided into two opposite but interconnected forces: Yin and Yang. For instance, the earth represents Yin, while the sky represents Yang, reflecting a universal concept. Specifically for human, the spirit is considered Yin, while the body is considered Yang. Health is believed to arise from the balance between Yin and Yang, both internally and within the surrounding environment. Additionally, individuals' health is influenced by the balance between different materials and the harmony between their 'Yin essence' and 'Yang spirit' [20].

Under the guidance of the general 'Yin Yang' theory, the most recognized definition of health is 'Ping ren' [21, 22, 27–32], which refers to a person in a healthy state with harmonious blood circulation, calm breathing, and steady pulse. TCM practitioners commonly view these indicators as signs of good health [19, 22, 24]. The concept of 'Yin Yang' aligns with another related phrase, 'He,' which emphasizes ‘harmony’ as the essence of health. 'He' represents the harmony between ‘Qi’ (energy in the broadest sense possible) and blood (important indicators of physical health in TCM) and the harmony of the spirit (associated with mental and emotional well-being). Both 'Yin Yang' and 'He' reflect characteristic philosophical thoughts in Chinese traditional culture [26, 30].

In the identified studies, the three most commonly used concepts under the 'Yin Yang' theory for defining and measuring health were as follows: ‘Xing Shen Tong Yi'(形神统一) [17–20, 22, 25–27, 31, 33, 35–37], 'Tian Ren He Yi'(天人合一) [17, 19, 20, 22, 25–28, 35, 37, 38] and 'Qi Qing'(七情) [17, 25, 27, 36, 37].

#### Xing Shen Tong Yi

'Xing Shen Tong Yi' means the unity of body and spirit, which can be specifically divided into two domains: 'xing' and 'shen'. The 'xing' domain refers to the body structure, such as the viscera, meridians, essence, blood, muscles, limbs, and bones [19], focusing on physical health. By summarizing the included studies, the following facets were identified: stamina, sleep, appetite and digestion, complexion, stool, mobility, self-care, discomfort, pain, urination, voice, and constitution. These indicators can be used to confirm whether a person is physically healthy or not.

The 'Shen' domain has a broader meaning, which refers to both the gods of nature (i.e. natural phenomena and laws) and the mental state of the human body (i.e. emotions, will, personality, memory, and perception) [22]. By summarizing the included studies, the following facets were identified: thinking, verbal expression, consciousness, spirit of the eyes, memory, concentration, fatigue, confidence, and satisfaction. 'Xing Shen Tong Yi' also reflects the basic view of holism in TCM that people are an organic integrity, and that the body and mind are closely interdependent [20].

#### Tian Ren He Yi

TCM emphasizes the unity of the human body and its relationship with the external environment [17, 27]. 'Tian Ren He Yi' emphasizes the harmony between humans and environment [17, 20, 35], which can be divided into two domains: natural environment and social environment.

In the 'natural environment' domain, TCM highlights the interconnectedness between humans and nature. Any changes or movements in nature will directly or indirectly affect the human body, such as seasonal climate changes, regional differences, leading to physiological discomfort or pathological changes [17]. This domain encompasses two facets: climate adaptation and adjustment, as well as dwelling conditions.

In the 'social environment' domain, TCM recognizes that humans are not only part of nature but also part of society. Health is influenced by various social factors, including political, economic, cultural, marriage, and interpersonal aspects. These factors will affect people's emotional changes, which can further impact the health of human. Common facets in this domain include socialization (e.g., communication, sex life, and loneliness), family (including family relationships, support, and conflicts), work (including relationships with colleagues and work performance), and economic conditions [39].

The concept of 'Tian Ren He Yi' is the direct embodiment of the relationship between human and environment. People's adaptation to the external environment is also the external embodiment of human health. Maintaining harmony between humans and the natural environment is of great importance [19, 20, 35].

#### Qi Qing

In TCM, the concept of 'Qi Qing' refers to the seven basic emotions: joy, anger, worry, pensiveness, grief, fear, and anxiety [17]. According to TCM, these emotions are closely linked to the five internal organs to our body: heart, liver, spleen, lung, and kidney [36, 37]. Each emotion corresponds to a specific organ, and when emotions become intense or unstable, they can disrupt the balance of ‘Qi’ (energy in the broadest sense possible) and blood, leading to physical problems. For example, anger affects the liver, joy affects the heart, pensiveness affects the spleen, worry affects the lung, and fear affects the kidney [17]. Therefore, maintaining emotional stability is essential for overall health and proper organ functioning.

Additionally, some of the definitions of HRQoL mentioned in the eligible studies may not fall within the purview of 'Yin Yang'. For instance, Confucianism emphasizes positive attitude and family relationships [18, 22], as well as the concept of ‘moderation’ to maintain a healthy state [22]. Taoism highlights conforming to nature and maintaining peace of mind without overthinking [22, 30, 31, 40]. Buddhism places importance on virtue and the theory of karma, where good behavior leads to positive outcomes, including physical health and mental satisfaction [18, 22].

### MM framework

**Fig. 3:**
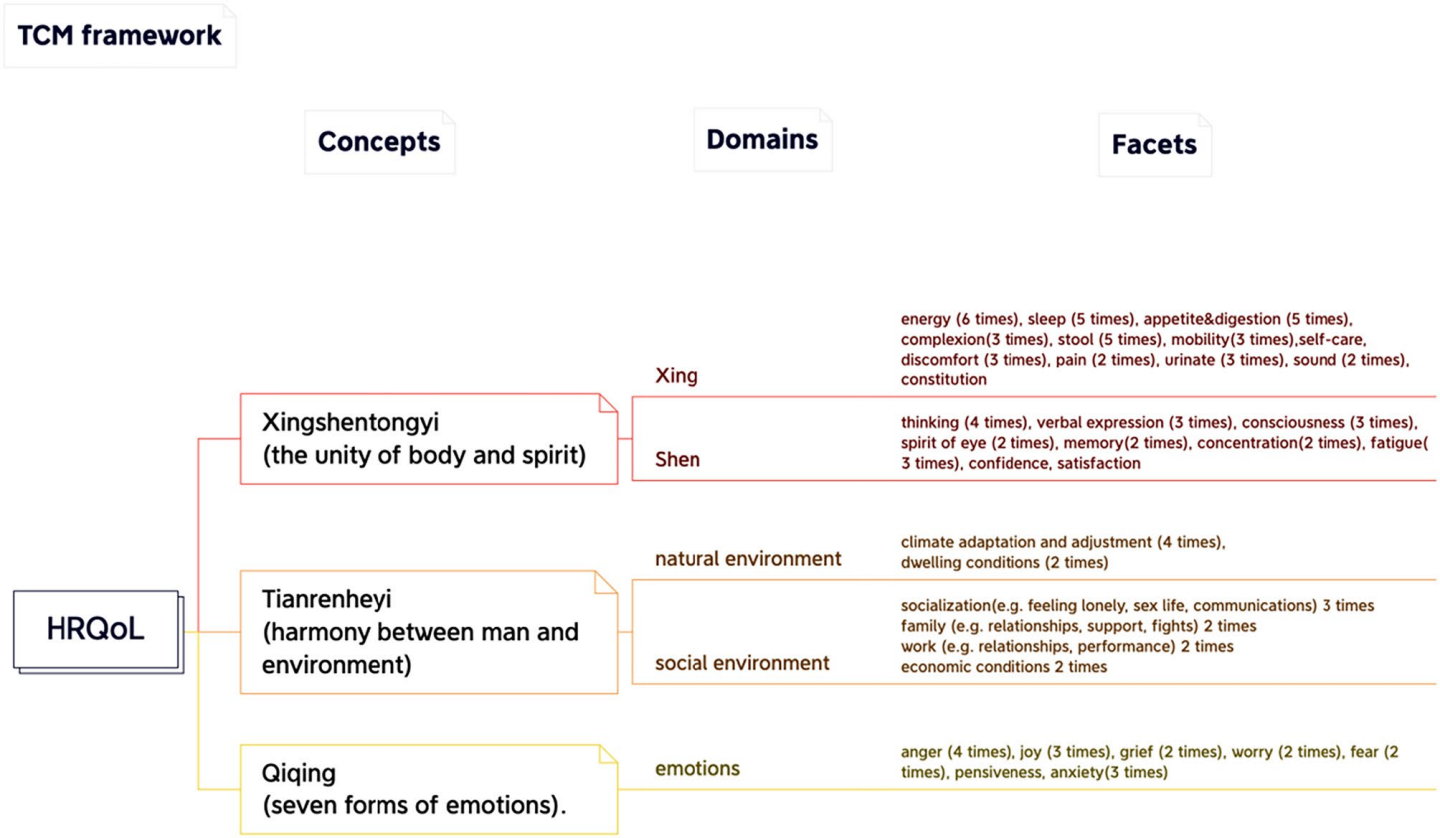
Traditional Chinese medicine framework

**Fig. 4:**
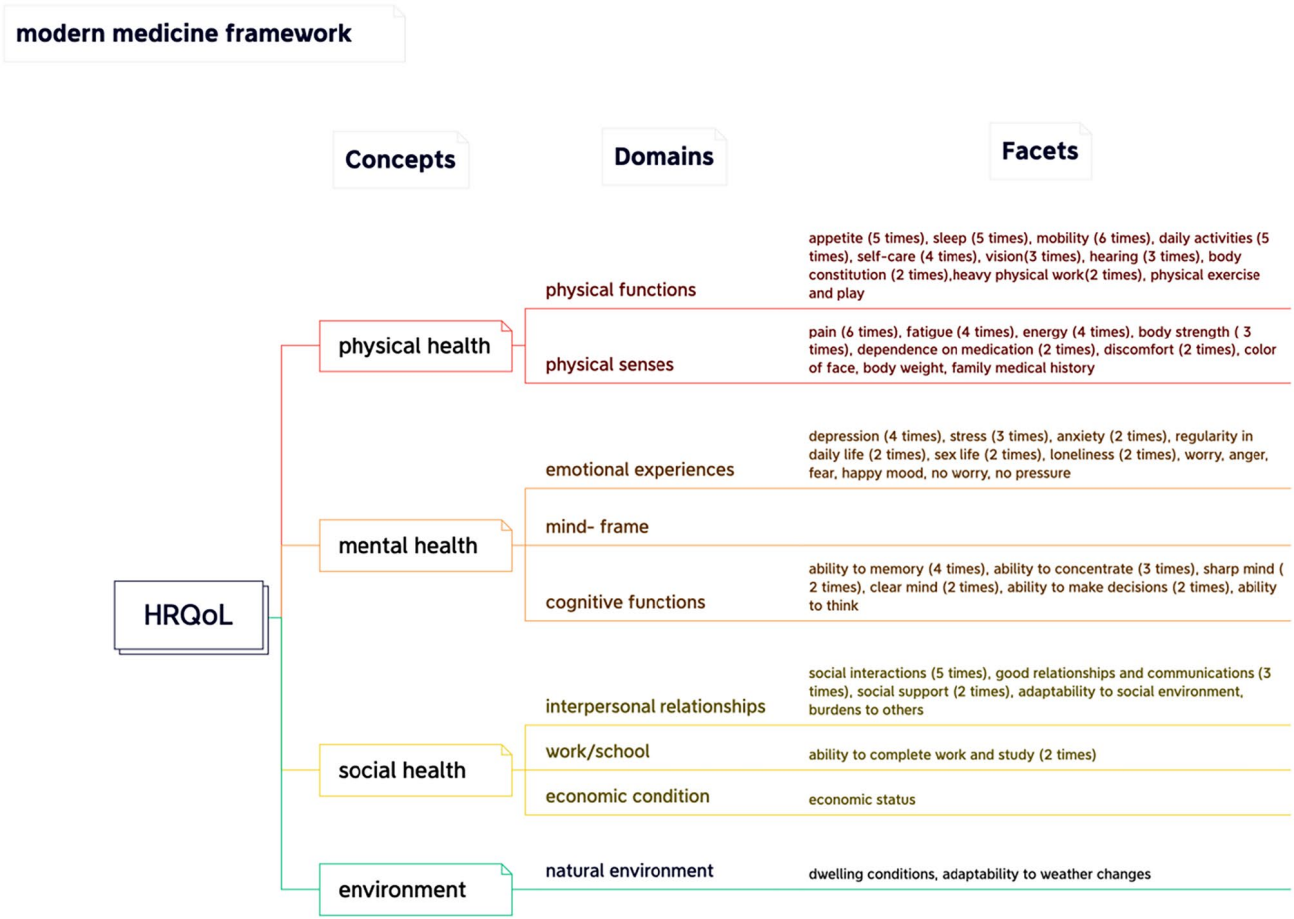
Modern medicine framework

From MM's perspective, two definitions were discussed. Firstly, one study adopted the definition from 'Ci Hai,' an authoritative Chinese dictionary [35]: ‘The state of well-developed organs and systems, normal functions, strong physique, full of energy, and high labor efficiency, which are usually measured through physical examination and various physiological indicators’ [41]. Secondly, most studies on HRQoL were based on the World Health Organization's (WHO) definition of ‘health’ [10, 18, 19]: ‘health is a complete state of physical, mental, and social well-being, not simply the absence of disease or infirmity’ [42]. The WHO definition of ‘health’ was not only used in defining HRQoL but also incorporated into the development of HRQoL instruments. Most studies defined HRQoL using the following three concepts: physical health, mental health, and social health, with some suggesting modifications. In the Chinese context, the concept of 'environment' such as dwelling conditions and changes in climate may be added [18, 21–23, 38]. The detailed framework results are shown in Figs. 3 and 4.

#### Physical health

The concept of 'physical health' can be divided into two domains: 'physical function' and 'physical senses'. In the domain of ‘physical function’, researchers focus on basic functions of body, such as appetite, sleep, mobility, daily activities, self-care, vision, hearing, body constitution, physical exercise, recreational activities, and heavy physical work. In the domain of ‘physical senses’, attention is given to discomfort, pain, and disease, reflected in facets such as pain, fatigue or tiredness, energy or vitality, body weight, dependence on medication, discomfort, complexion, body strength, and family medical history.

#### Mental health

'Mental health' consists of three domains: emotional experiences, mind-frame, and cognitive functions. In the domain of ‘emotional experiences’, common facets include depression, stress, anxiety, regularity in daily life, sex life, loneliness, worry, anger, fear, happy mood, no worry, and no pressure. The ‘mind-frame’ domain encompasses facets like self-confidence, morality, positive attitude, peace, breadth of mind, and sense of life satisfaction. Lastly, the ‘cognitive function’ domain includes facets such as memory, concentration ability, sharp mind, clear mind, decision-making ability, and thinking ability.

#### Social health

'Social health' can be divided into three domains: interpersonal relationships, work or school, and economic condition. ‘Interpersonal relationships’ emphasize the ability to communication with others and the adaptability to the environment. It includes the following facets: social interactions, good relationships and communications with others (e.g. friends, family and colleagues), social support, adaptability to social environment and burdens to others. The ‘work or school’ domain concerns task completion and performance in studies or job. Additionally, ‘economic conditions’ are also a crucial component of overall mental health.

#### Environment

Apart from the three fundamental WHO health concepts, namely physical, mental, and social health, the concept of 'environment' has also been recognized as an important factor in the included studies [18, 21–23, 38]. This domain encompasses the ability to adapt to changes in weather and living conditions.

### Overlap between TCM and MM

Both TCM and MM break down their concepts of HRQoL into 'domains', as depicted in Figs. 3 and 4. These domains demonstrate significant overlap between TCM and MM perspectives. They encompass intrapersonal (such as physical and emotional aspects) and interpersonal domains (such as relationships and work).

**Fig. 5:**
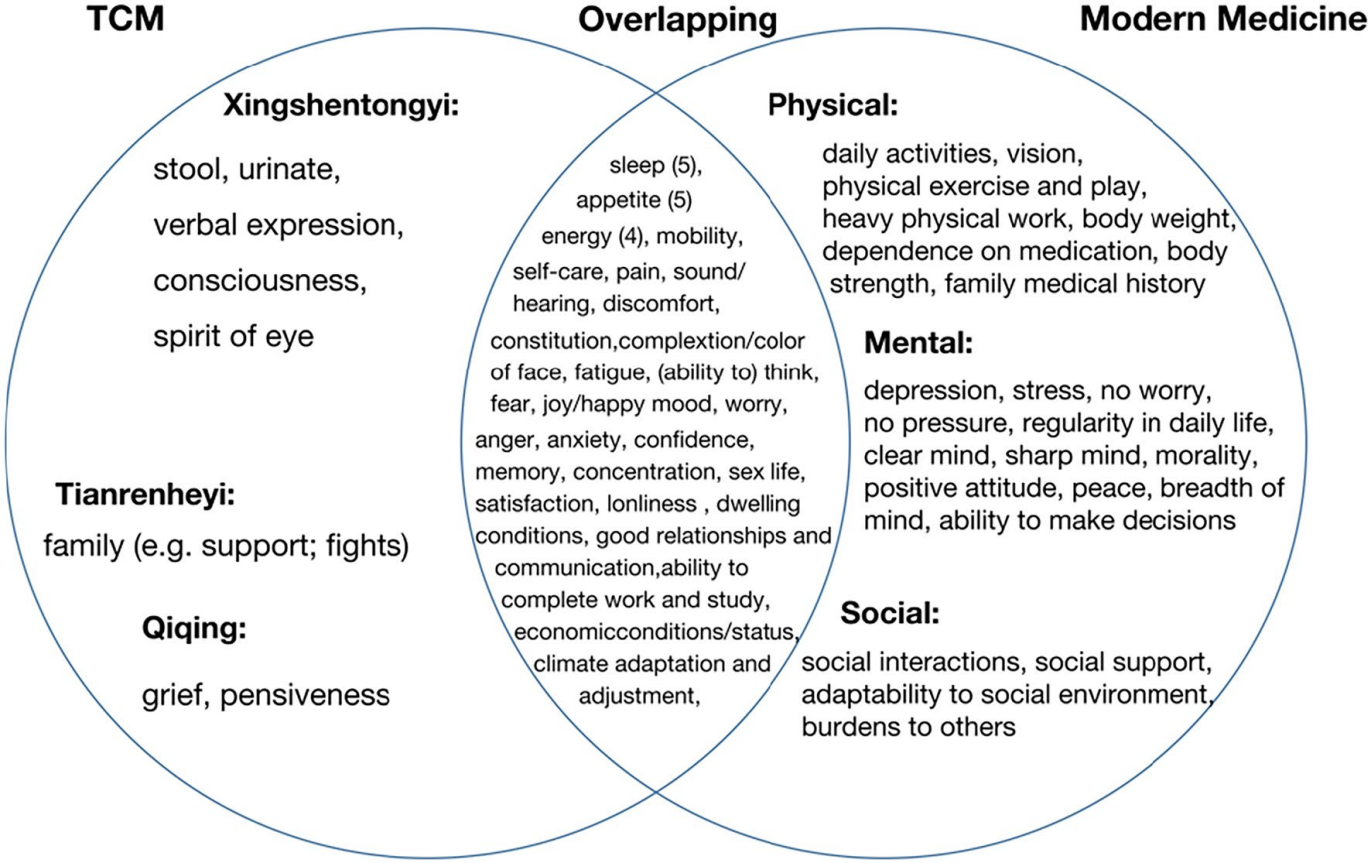
Intersection diagram of the facets of TCM and the MM frameworks

When these domains are operationalized into survey questions, the resulting 'facets' further reveal the similarities between TCM and MM. Figure 5 illustrates that many facets share common wording. Among the 59 facets identified, 28 were addressed in both TCM and MM, 9 were specific to TCM, and 22 were specific to MM. Among the shared facets, we found that 'appetite', 'sleep' and 'energy' were the most frequently mentioned facets in both frameworks.

## Discussion

This paper aims to explore and synthesize perceptions of HRQoL within a Chinese cultural setting. Our systematic review of relevant research reveals that two distinct perspectives existed in defining the conceptual frameworks of HRQoL in China: TCM and MM. To provide a comprehensive overview of these perspectives, we summarized the conceptual frameworks of both perspectives.

Firstly, it is challenging to define HRQoL. Mayo et al. proposed distinguishing between QoL and HRQoL [43]. However, Chinese studies often use the WHO's definition of 'health' instead of Mayo's HRQoL definition. The Chinese studies used the WHO’s definition of 'health' for defining HRQoL and also used the WHO definition of 'health' for the development of HRQoL instruments [10, 18, 19]. Meanwhile, examining HRQoL from TCM perspective reveals a focus on broader QoL aspects. TCM's emphasis on 'a balance between oneself and the environment' relates more to QoL than the narrow 'impact of disease and treatment' in the HRQoL definition from Mayo.

The two frameworks exhibit certain differences. On the level of concepts, TCM takes a holistic view, emphasizing the interconnectedness and interaction between body and mind, while MM does not. TCM framework uses terms like 'unity' and 'harmony' to underscore this holistic perspective and interaction, considering the inseparability of body and spirit [34]. By contrast, MM seems to be influenced by the theory of 'mind–body dualism,' separating the mind and body. According to the MM framework, individuals consist of two separate substances: body and mind, which can be described independently. As a result of these fundamental differences, the descriptions of HRQoL from these two frameworks consistently differ. Notably, though the HRQoL conceptual framework of TCM emphasizes the unity of health, instruments developed based on this conceptual framework often do not fully capture this unity. For instance, the Chinese Medicine Quality of Life-11 Dimensions (CQ-11D) emphasizes the unity of body and spirit but it still measures ‘body and ‘mind’ as two independent dimensions [33]. Therefore, we have observed that the idea of 'holistic' is difficult to embody in TCM instruments. This can be seen in Fig. 3.

Differences in specific facets describing HRQoL are also evident (Fig. 5). The TCM framework incorporates unique diagnostic indicators, many of which are specific to TCM-related 'symptoms' such as 'defecation' and 'spirit of eyes'. In contrast, MM focuses on measurable physical phenomena like 'vision' and 'weight'. Our analysis highlights two main reasons for these differences. Firstly, TCM places emphasis on 'image thinking' [44, 45], which involves intuitively grasping the abstract meaning of the world and its universal connections through intuition, metaphor, symbol, association, and analogy [45–47]. TCM employs various 'images,' such as tongue image, pulse image, and syndrome image [47]. Based on this idea, TCM generally collects patients' symptoms through ‘making observations, listening to breathing, asking about symptoms and taking the pulse (the four fundamental methods for diagnosis in TCM)’ for analysis and syndrome differentiation. However, MM focuses on analysing pathological mechanisms using objective and measurable examination results [48]. Secondly, the discrepancy in specific facets is related to a well-accepted concept known as sub-health, which refers to a state between illness and health [49, 50]. Sub-health is characterized by experiencing different types of symptoms, both physical (e.g., pain, discomfort, fatigue) and mental (e.g., negative emotions, poor memory, inattention), for more than three months without any clear clinical attribution [51, 52]. In MM, there is no specific diagnose for such health status specific diseases when individuals experience prolonged fatigue despite normal clinical indicators. However, TCM theories have significantly contributed to the widespread recognition and acceptance of the sub-health concept.

Despite some differences, these two conceptual frameworks also share similarities. As mentioned before, while each framework has its unique facets, there is a significant overlap between them, as shown in the middle section of Fig. 5. This overlap may be inherent to TCM and MM, but can also be the result ‘borrowing’ aspect of TCM into MM and vice versa. The integration of these two frameworks can be observed in Fig. 5. MM also acknowledges the interconnectedness and interaction of body and mind when considering HRQoL. For instance, in the MM framework, there are facets that resemble TCM facets, such as 'complexion', 'energy', and 'constitution'. Similarly, in the TCM framework, facets emphasized by MM seem to be borrowed, like 'self-care' and 'mobility'. Among the shared facets, we found that 'appetite', 'sleep' and 'energy' were the most frequently mentioned facets in both frameworks. These can be considered vital elements in how people in China define HRQoL. The importance of 'appetite' can be attributed to various factors, including China’s historical experiences with varying food supply and the social aspect of eating [53]. For example, sharing meals serves as a common way for people to establish and express connections with one another in China. Additionally, 'sleep' was also recognized as an essential facet in most HRQoL instruments within the Chinese cultural setting. TCM regards 'sleep' as crucial for well-being and preventing illness [54–56]. Compared to TCM, MM only recently acknowledges that sleep is vital for cognitive function, emotion, memory, endocrine balance, and immunity [57]. Furthermore, 'energy' was also frequently mentioned and covered by the two frameworks. In Chinese culture, 'energy' is closely related to the concept of 'Qi' (also spelled chi) in TCM [58, 59]. Qi is believed to exist in all things, including air, water, food, and sunlight, and is often translated as 'vital energy'—a fundamental substance that builds and sustains the body [60]. Furthermore, it is important to recognize that concepts from two types of frameworks are divided into 'domains', and these domains exhibit considerable overlap (Figs. 3, 4). These figures demonstrate that both frameworks describe HRQoL from four aspects: physical, mental, social, and adaptability to the natural environment. Although the theoretical basis and classification of the two frameworks may differ, the content is quite similar.

Although there are many overlapping facets between two frameworks, we still chose to describe HRQoL using two separate frameworks rather than combining them into one in this article. There are three main reasons: firstly, the original intention was to provide a comprehensive understanding of HRQoL in Chinese cultural. After a systematic literature review and content classification, we found that presenting two conceptual frameworks from different perspectives better represents the definition of HRQoL in China. Secondly, both frameworks have unique facets that cannot be merged. TCM includes facets such as 'urinate', 'stool', and 'spirit of eye', whereas the MM-based framework includes 'body weight', 'dependence on medication', and 'burdens to others'. Thirdly, even for overlapping facets, they have different classifications in each framework due to distinct theories. For example, 'emotions' holds different positions in the two frameworks. In TCM, it is considered a 'concept' contributing to overall health, ranked alongside 'the unity of form and spirit' and 'the unity of man and nature'. In MM, 'emotion' is a domain under the concept of 'mental health'. This divergence arises from MM viewing emotions as only a component of mental health, while TCM recognizes that emotions not only affect an individual's mental state but also have specific relationships with different organs in the body. As a result, TCM emphasizes the importance of maintaining emotional stability and avoiding extreme emotions for good health, leading us to list it separately in the TCM framework.

However, it would be beneficial to integrate these frameworks in future research. This integration aligns with the strategy of 'integrating Chinese and modern medicine', which is widely practiced in clinical practice in China. Integrative medicine, with its efficacy and complementary advantages, has emerged as one of the major medical systems alongside MM and TCM [61]. In Chinese clinical guidelines, it's common to find a combination of conventional disease diagnoses and traditional syndromes. While there are globally recognized standards for diagnosing diseases (such as the International Classification of Diseases, ICD-10), there's also an emphasis on identifying syndromes that reflect traditional Chinese characteristics [61]. Supported by the government policy, 'integrating Chinese and modern medicine' will continuously be the trend and our results also support this conclusion. It is evident that in the Chinese context, the definition of HRQoL cannot be adequately captured from a single perspective alone. Therefore, future research should aim to integrate the two HRQoL frameworks to provide a more comprehensive understanding.

The conclusion evokes questions whether we need a specific Chinese HRQoL instrument or if existing ones are sufficient. While instruments focused on 'Integrating Chinese and Modern medicine' naturally differ from Western-developed ones, it's important to assess if they truly benefit Chinese researchers internationally. We need to consider if a 'real Chinese HRQoL instrument' would be valuable outside of China, which could potentially devalue research from China and contradict the findings of the present study. Despite China having two well-developed HRQoL concepts, one possibly unique to China, they seem quite similar in terms of their main ideas.

As mentioned in the methods section, a limitation is the absence of a quality evaluation for the included articles. The second limitation is the inherent subjectively in classifying the concepts, domains and facets. In order to address this, we will employ 'concept mapping' in the future to provide a more objective exploration of the overlap in dimensions and facets between MM and TCM. The third limitation is that our research only included the general population and generic questionnaires. Patient-Reported Outcomes (PROs) are often diseases specific, such as cancer [62], but we were limited in the use of them due to the variance in patient groups, which would in turn impact how HRQoL is defined. However, this does not imply that PROs cannot be investigated. One potential solution could be to categorize specific disease groups. For instance, several authors have examined the applications and characteristics of PRO instruments as primary and secondary outcomes in randomized clinical trials in China [63], as well as the use of PROs in clinical trials of TCM [64] to promote and standardize PROs in China.

## Conclusion

The present study is the first systematic review summarizing the conception of HRQoL in the Chinese cultural setting. We found two distinct frameworks of HRQoL exist in China, each basing on its own unique theory: MM and TCM, but arriving at some similar concepts, domains and facets.

### Supplementary Information

Below is the link to the electronic supplementary material.

Supplementary file1 (DOCX 19 KB)

Supplementary file2 (XLSX 23 KB)

Supplementary file3 (XLSX 26 KB)

Supplementary file4 (XLSX 15 KB)

Supplementary file5 (DOCX 187 KB)

Supplementary file6 (DOCX 33 KB)

Supplementary file7 (DOCX 19 KB)

## Data Availability

The data supporting the findings of this study are accessible in both the article and its supplementary material.
