# Supplementary material for: Differences and common ground in the frameworks of health-related quality of life in traditional Chinese medicine and modern medicine: a systematic review
Source: Qual Life Res. 2024 May 13;33(7):1795–806. doi: 10.1007/s11136-024-03669-1 (PMC11176225; doi:10.1007/s11136-024-03669-1)
Supplement: Supplementary file 1 — Supplementary file1 (DOCX 19 KB) [file 11136_2024_3669_MOESM1_ESM.docx]

**Information**

**Article title:** Differences and Common Ground in the Frameworks of Health-Related Quality of Life in Traditional Chinese Medicine and Modern Medicine: A Systematic Review

**Journal name:** Quality of life Research

**Author names:** Yifan Ding; Zhuxin Mao; Nan Luo; Zhihao Yang; Jan van Busschbach

**Affiliation of the corresponding author:** Erasmus MC, University Medical Center Rotterdam, Department of Psychiatry, Rotterdam, the Netherlands

**E-mail address of the corresponding author:** y.ding@erasmusmc.nl

**Appendix 1: Searching strategy (English Datebase)**

**Embase :**

('Chinese'/de OR 'China'/exp OR (china* OR chines* OR mandarin* OR cantones* OR hong-kong):ab,ti,kw) **AND** ('quality of life assessment'/de OR (((quality-of-lif* OR life-qualit* OR life-satisfact* OR resident-satisfact* OR QOL OR HRQOL OR hqol OR h-qol OR hr-qol) NEAR/6 (assess* OR survey* OR questionna* OR index* OR measur* OR instrument* OR tool* OR measur* OR focus-group* OR interview* OR content* OR concept* OR dimension* OR domain* OR aspect* OR facet* OR connotation* OR definition* OR framework* OR construct* OR structure*))):ab,ti,kw) NOT ((animal/exp OR animal*:de OR nonhuman/de) NOT ('human'/exp)) NOT ([Conference Abstract]/lim OR [preprint]/lim)

**Medline:**

(exp China/ OR (china* OR chines* OR mandarin* OR cantones* OR hong-kong).ab,ti,kf.) AND ((((quality-of-lif* OR life-qualit* OR life-satisfact* OR resident-satisfact* OR QOL OR HRQOL OR hqol OR h-qol OR hr-qol) ADJ6 (assess* OR survey* OR questionna* OR index* OR measur* OR instrument* OR tool* OR measur* OR focus-group* OR interview* OR content* OR concept* OR dimension* OR domain* OR aspect* OR facet* OR connotation* OR definition* OR framework* OR construct* OR structure*))).ab,ti,kf.) NOT (exp animals/ NOT humans/) NOT (news OR congres* OR abstract* OR book* OR chapter* OR dissertation abstract*).pt.

**Cochrane **:**

((china* OR chines* OR mandarin* OR cantones* OR hong-kong):ab,ti,kw) **AND** (((((quality NEXT/1 of NEXT/1 lif*) OR (life NEXT/1 qualit*) OR (life NEXT/1 satisfact*) OR (resident NEXT/1 satisfact*) OR QOL OR HRQOL OR hqol OR (h NEXT/1 qol) OR (hr NEXT/1 qol)) NEAR/6 (assess* OR survey* OR questionna* OR index* OR measur* OR instrument* OR tool* OR measur* OR (focus NEXT/1 group*) OR interview* OR content* OR concept* OR dimension* OR domain* OR aspect* OR facet* OR connotation* OR definition* OR framework* OR construct* OR structure*))):ab,ti,kw)

**Web of Science* :**

TS=(((china* OR chines* OR mandarin* OR cantones* OR hong-kong)) AND (((((quality NEAR/1 of NEAR/1 lif*) OR (life NEAR/1 qualit*) OR (life NEAR/1 satisfact*) OR (resident NEAR/1 satisfact*) OR QOL OR HRQOL OR hqol OR (h NEAR/1 qol) OR (hr NEAR/1 qol)) NEAR/5 (assess* OR survey* OR questionna* OR index* OR measur* OR instrument* OR tool* OR measur* OR (focus NEAR/1 group*) OR interview* OR content* OR concept* OR dimension* OR domain* OR aspect* OR facet* OR connotation* OR definition* OR framework* OR construct* OR structure*)))) NOT ((animal* OR rat OR rats OR mouse OR mice OR murine OR dog OR dogs OR canine OR cat OR cats OR feline OR rabbit OR cow OR cows OR bovine OR rodent* OR sheep OR ovine OR pig OR swine OR porcine OR veterinar* OR chick* OR zebrafish* OR baboon* OR nonhuman* OR primate* OR cattle* OR goose OR geese OR duck OR macaque* OR avian* OR bird* OR fish*) NOT (human* OR patient* OR women OR woman OR men OR man))) AND DT=(Article OR Review OR Letter OR Early Access)

**Google Scholar**

China|chinese “quality life assessment|questionnaire”

*Science Citation Index Expanded (1975-present) ; Social Sciences Citation Index (1975-present) ; Arts & Humanities Citation Index (1975-present) ; Conference Proceedings Citation Index- Science (1990-present) ; Conference Proceedings Citation Index- Social Science & Humanities (1990-present) ; Emerging Sources Citation Index (2015-present)

** Manually deleted abstracts from trial registries

**Searching Strategy (Chinese Database):**

**CNKI：**

(TI=(('量' + '问' + '调查') * ('表' + '卷') + '工具') and AB=('生命质量' + '生存质量' + '

生活质量' + '主观健康' + '自评健康' + 'PRO' + 'HROoL' + 'QoL') and AB=('制' * ('

定' + '订') + ( '编' + '研') * '制' + '测试' + '检验' + '研究' + '分析' + '修订' + '建立' + '

设计' + '搭建')) OR (TKA=('健康概念' + '健康框架' + '健康维度' + '健康内涵' + '健

康定义' + '健康内容' + '健康结构') and TKA=('中国' + '中华' + '中医药' + '东方' + '

华夏' + '传统' + '传统医学' + '中医')) OR (TKA=('健康概念' + '健康理解' + '健康定

义' + '健康内容' + '健康内涵') and TKA=(('定性' + '质性') * ('研究' + '访谈')))

**Wanfang:**

(题名:((“量” or “问” or “调查”) and (“表” or “卷” ) or “工具” ) AND 摘要:(“生命质

量” or “生存质量” or “生活质量” or “主观健康” or “自评健康” or “PRO” or “HROoL”

or “QoL”) AND 摘要:(“制” and (“定” or “订”) or (“编” or “研”) and “制” or “测试” or

“检验” or “研究” or “分析” or “修订” or “建立” or “设计” or “搭建”)) OR ((题名或

关键词:(“健康概念” or “健康框架” or “健康维度” or “健康内涵” or “健康定义” or

“健康内容” or “健康结构”) AND 题名或关键词:(“中国” or “中华” or “中医药” or

“东方” or “华夏” or “传统” or “传统医学” or “中医”)) OR (摘要:(“健康概念” or “健

康框架” or “健康维度” or “健康内涵” or “健康定义” or “健康内容” or “健康结构”)

AND 摘要:(“中国” or “中华” or “中医药” or “东方” or “华夏” or “传统” or “传统医

学” or “中医”))) OR ((题名或关键词:("健康概念" or "健康理解" or "健康定义" or "

健康内容" or "健康内涵") AND 题名或关键词:(("定性" or "质性") and ("研究" or "

访谈"))) OR(摘要:("健康概念" or "健康理解" or "健康定义" or "健康内容" or "健康

内涵") AND 摘要:(("定性" or "质性") and ("研究" or "访谈"))))

**Weipu：**

(T=((“量” + “问” + “调查”) * (“表” + “卷”) + “工具”) and U=(“生命质量” + “生存质量”

+ “生活质量” + “主观健康” + “自评健康” + “PRO” + “HROoL” + “QoL”) and U=(“制”

* (“定” + “订”) + (“编” + “研”) * “制” + “测试” + “检验” + “研究” + “分析” + “修订” +

“建立” + “设计” + “搭建”)) OR (U=(“健康概念” + “健康框架” + “健康维度” + “健康

内涵” + “健康定义” + “健康内容” + “健康结构”) and U=(“中国” + “中华” + “中医药”

+ “东方” + “华夏” + “传统” + “传统医学” + “中医”)) OR (U=(“健康概念” + “健康理

解” + “健康定义” + “健康内容” + “健康内涵”) and U=((“定性” + “质性”) * (“研究” +

“访谈”)))
