# Supplementary material for: Differences and common ground in the frameworks of health-related quality of life in traditional Chinese medicine and modern medicine: a systematic review
Source: Qual Life Res. 2024 May 13;33(7):1795–806. doi: 10.1007/s11136-024-03669-1 (PMC11176225; doi:10.1007/s11136-024-03669-1)
Supplement: Supplementary file 5 — Supplementary file5 (DOCX 187 KB) [file 11136_2024_3669_MOESM5_ESM.docx]

**Appendix 5:** Additional Explanations of Traditional Chinese Medicine (TCM) Terminology

We have observed that certain terms within Traditional Chinese Medicine (TCM) are abstract. Due to the word limit, we did not give sufficient explanations in the main text of first edition. As a result, we have provided more information in this appendix 5 for better understanding. Within the TCM framework, we offer supplementary insights into three key concepts (as shown in the figure): Xing shen tong yi; Tian ren he yi; Qi qing.


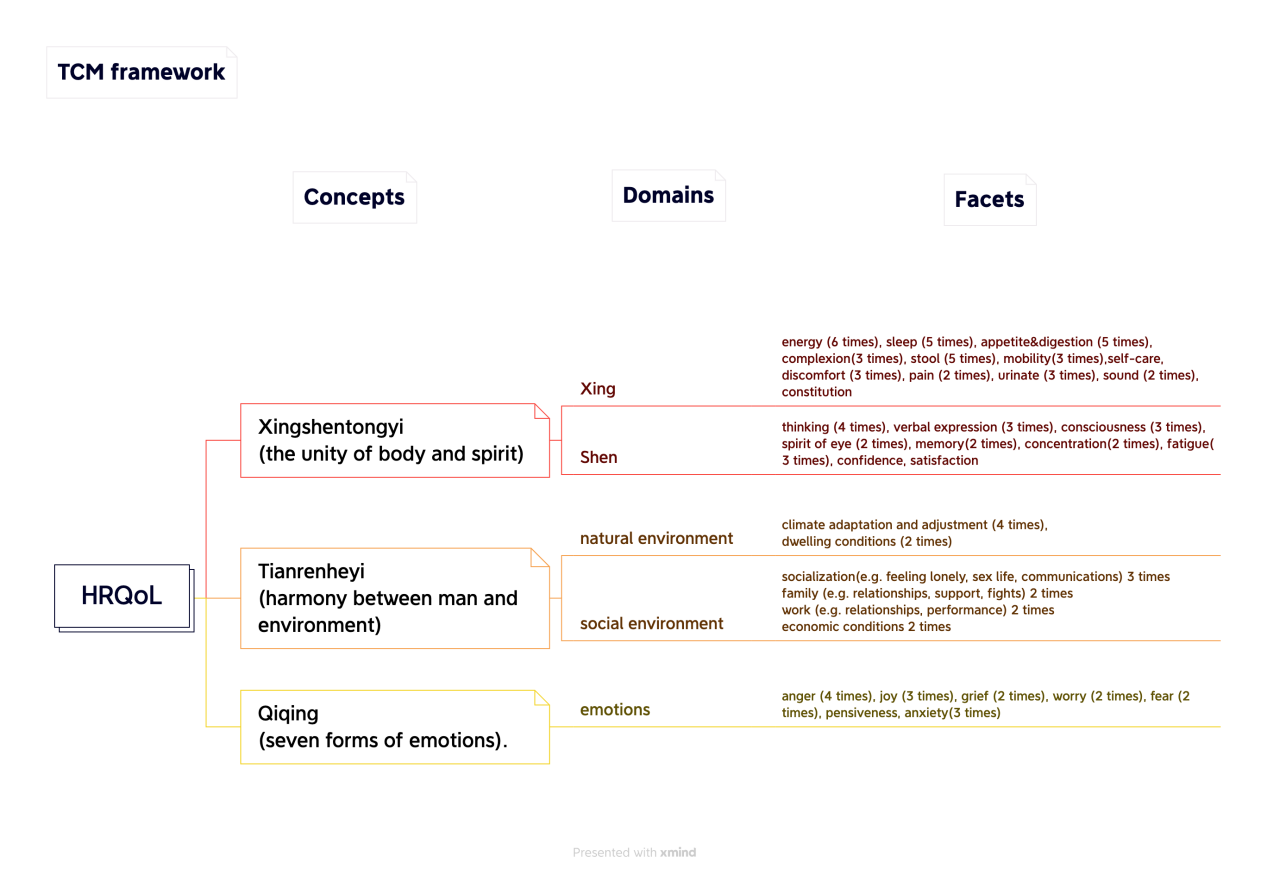


- **‘Xing shen tong yi’** (形神统一) is a concept from TCM and Chinese philosophy that can be translated to ‘Unity of Body and Spirit’. ‘Xing’ represents the ‘body’ and ‘Shen’ represents the ‘spirit’. This concept highlights the interconnectedness of physical and mental aspects of a person's well-being. In TCM, it suggests that a person's physical health is closely linked to their mental and emotional well-being, and vice versa. When the body (Xing) and spirit (Shen) are in harmony and balance, it is believed to contribute to overall health and vitality. Conversely, imbalances or disharmony between the two can lead to various health issues. Balancing both physical and mental aspects is a fundamental principle in TCM.
- **‘Tian ren he yi’** (天人合一) is often translated as the ‘Harmony between Man and Environment.’ This concept is closely associated with TCM, particularly Daoism (Taoism) and Confucianism. In the context of ‘Tian ren he yi’, ‘Tian’ represents ‘environment’, and ‘ren’ represents ‘man’. The concept emphasizes the idea that human beings are interconnected with and influenced by the natural world, and there is an essential unity between humans and the broader environment. It suggests that human well-being and harmony can be achieved by aligning one's life and actions with the rhythms and principles of nature. This concept encourages living in balance with the natural world, respecting the environment, and recognizing that humans are not separate from nature but rather an integral part of it.In a broader sense, ‘Tian ren he yi’ promotes an ecological and harmonious perspective on the relationship between humans and the environment, advocating for sustainability and a lifestyle that is in accord with the natural world.
- **‘Qi qing’** (七情) is often translated as the ‘Seven Emotions’. TCM believes that there are seven basic emotions for human: joy, anger, worry, pensiveness, grief, fear and anxiety. The seven emotions theory stresses that emotion and body are linked and mutually affected. It is believed that the human body is an organic whole centered on the five internal organs: heart, liver, spleen, lung and kidney. Emotional activities are closely linked to the essence of these five internal organs. According to the ancient Chinese medicine records, the five internal organs store essence, which is refined into the body's vital energy (qi) and its overall balance. The movement of qi responds to the external environment and gives rise to emotional activities. Consequently, each of the five internal organs is associated with specific emotional activities: the liver is linked to anger, the heart to joy, the spleen to pensiveness, the lung to worry, and the kidney to fear. Excessive or imbalanced emotions can disrupt the flow of qi and lead to health issues. Overall, in TCM, maintaining emotional stability plays an crucial role in keeping health. Emotional instability can lead to physical problems and affect the normal functioning of the five internal organs.
