# Supplementary material for: Differences and common ground in the frameworks of health-related quality of life in traditional Chinese medicine and modern medicine: a systematic review
Source: Qual Life Res. 2024 May 13;33(7):1795–806. doi: 10.1007/s11136-024-03669-1 (PMC11176225; doi:10.1007/s11136-024-03669-1)
Supplement: Supplementary file 6 — Supplementary file6 (DOCX 33 KB) [file 11136_2024_3669_MOESM6_ESM.docx]

| **Section and Topic** | **Item #** | **Checklist item** | **Location where item is reported** |
| --- | --- | --- | --- |
| **TITLE** | | |  |
| Title | 1 | Identify the report as a systematic review.  Differences and Common Ground in the Frameworks of Health-Related Quality of Life in Traditional Chinese Medicine and Modern Medicine: A Systematic Review | p1 |
| **ABSTRACT** | | |  |
| Abstract | 2 | See the PRISMA 2020 for Abstracts checklist. | p2 |
| **INTRODUCTION** | | |  |
| Rationale | 3 | Describe the rationale for the review in the context of existing knowledge.  It is described in the Introduction part. | p4 |
| Objectives | 4 | Provide an explicit statement of the objective(s) or question(s) the review addresses.  This study aims to systematically review published studies that describe the theoretical and operationalised conceptualization of HRQoL. Our objective is to explore and synthesize perceptions of HRQoL within a Chinese cultural setting. | p4 |
| **METHODS** | | |  |
| Eligibility criteria | 5 | Specify the inclusion and exclusion criteria for the review and how studies were grouped for the syntheses.  **Inclusion criteria:** Our search was focused on publications related to the following topics: a) reporting HRQoL measures that were developed in a Chinese cultural setting; b) discussing the definition of HRQoL in Chinese cultural setting or constructing a conceptual framework of HRQoL specifically to Chinese culture; c) conducting qualitative interviews to explore Chinese people's understandings of HRQoL.  **Exclusion criteria:** a) studies focused on a specific disease; b) studies focused on a particular population (e.g., elderly or specific region); c) studies utilizing existing instruments such as SF-36, EQ-5D, etc.; d) studies focused on other constructs (e.g., happiness, well-being, life satisfaction).  **Grouping process:** For studies focusing on a general definition of HRQoL, they introduced the foundational theory of TCM and supplied potential corroborative evidence for TCM and laid the foundation upon which TCM was developed. In light of these foundational theory, we extracted frequently- mentioned health concepts from the definitions as the cornerstone of the initial level (concept-level) of our framework. Subsequently, with the essence and comprehensions of these three concepts, we proceeded to construct the secondary level (domain-level) of our framework. For studies providing detailed descriptive system, we extracted all items referenced within each framework or instrument. The extracted items (see Appendix 3) were grouped into the third level (facet-level) and categorized each facets into specific domains. The frequency of each facet was recorded (see Appendix 4). During the grouping process, we referred to the existing categories of descriptive systems as found in papers because most of them were already grouped by the designers of frameworks or instruments. If facets were classified into different groups in different frameworks or instruments, we made the decision ourselves, according to our understandings. | p5 |
| Information sources | 6 | Specify all databases, registers, websites, organisations, reference lists and other sources searched or consulted to identify studies. Specify the date when each source was last searched or consulted.  We conducted a comprehensive search across three Chinese databases (CNKI, Weipu, and Wanfang) and four English databases (EMBASE, MEDLINE, Web of Science, and Cochrane), in addition to a restricted search on Google Scholar. We conducted a comprehensive review of various publication types, encompassing original research, reviews, commentaries, and dissertations. However, conference reports were excluded from our selection. Notably, there were no constraints based on publication date other than the specified end date (March 3rd, 2022). | p6 |
| Search strategy | 7 | Present the full search strategies for all databases, registers and websites, including any filters and limits used.  Both of detailed Chinese and English search strategies are presented in the **Appendix 1**. The Chinese search strategy was developed through an internal discussion by the four Chinese researchers in team. The English strategy was developed after the consultation with the librarian in Erasmus Medical Centrum, the Netherlands and the internal discussion of the whole research team. | p6 |
| Selection process | 8 | Specify the methods used to decide whether a study met the inclusion criteria of the review, including how many reviewers screened each record and each report retrieved, whether they worked independently, and if applicable, details of automation tools used in the process.   - Two bilingual reviewers (YD and ZM) reviewed the titles and abstracts for compliance with the eligibility criteria and their relevance for inclusion. In consultation with the review team (ZM, ZY, and NL), the first author (YD) independently reviewed the resulting full-text articles. The review team included one person with methodological experience and two persons with expertise on the topic. If there were differences in opinion about eligibility, these were resolved by mutual agreement, and if this was not reached, an external opinion was sought (JB). - automation tool used was EndNote X9. | p6-p7 |
| Data collection process | 9 | Specify the methods used to collect data from reports, including how many reviewers collected data from each report, whether they worked independently, any processes for obtaining or confirming data from study investigators, and if applicable, details of automation tools used in the process.   - According to the searching results, the key information of all eligible studies were extracted by the first author (YD), including the title, author, publish year, region, study aim, methods, and results (see **Appendix 2**). - During the grouping process, we referred to the existing categories of descriptive systems as found in papers because most of them were already grouped by the designers of frameworks or instruments. If facets were classified into different groups in different frameworks or instruments, we made the decision ourselves, according to our understandings.The grouping was independently conducted by two reviewers (YD and ZM), and any inconsistencies were resolved through internal team discussions. - The tool used was EXCEL. | p7 |
| Data items | 10a | List and define all outcomes for which data were sought. Specify whether all results that were compatible with each outcome domain in each study were sought (e.g. for all measures, time points, analyses), and if not, the methods used to decide which results to collect.  See **Appendix 2**. Appendix 2 is the data extraction table, including the title, author, publish year, region, study aim, methods, and results of all eligible papers. | p7 |
|  | 10b | List and define all other variables for which data were sought (e.g. participant and intervention characteristics, funding sources). Describe any assumptions made about any missing or unclear information.  Our research was not related to this problem. | Not applicable |
| Study risk of bias assessment | 11 | Specify the methods used to assess risk of bias in the included studies, including details of the tool(s) used, how many reviewers assessed each study and whether they worked independently, and if applicable, details of automation tools used in the process.  We did not explicitly perform any assessments using standardized criteria or tools for the studies included. This decision was driven by the primary goal of our study, which was to comprehensively gather relevant papers. However, it's worth noting that we did encounter certain papers from Chinese databases that included non-academic content, such as the article with the title '20 New Concepts of Healthy Living.' Nevertheless, we did take the quality of these studies from Chinese databases into account during our analysis. This consideration involved a qualitative assessment that considered factors such as study design, methodology, and the presence of bias or limitations. These factors were factored into our interpretation of the results. | Not applicable |
| Effect measures | 12 | Specify for each outcome the effect measure(s) (e.g. risk ratio, mean difference) used in the synthesis or presentation of results.  Our research was not related to this problem. | Not applicable |
| Synthesis methods | 13a | Describe the processes used to decide which studies were eligible for each synthesis (e.g. tabulating the study intervention characteristics and comparing against the planned groups for each synthesis (item #5)).  Appendix 2 is the data extraction table, including the title, author, publish year, region, study aim, methods, and results of all eligible papers. After reviewing all eligible articles, we categorized them into two groups based on their content: definition and framework. All studies describe HRQoL from two perspectives: Traditional Chinese Culture and Modern Medicine. | p7 |
|  | 13b | Describe any methods required to prepare the data for presentation or synthesis, such as handling of missing summary statistics, or data conversions.  We aimed to create two consolidated conceptual frameworks by combining all facets, domains, and concepts. | p7 |
|  | 13c | Describe any methods used to tabulate or visually display results of individual studies and syntheses.  Each study was presented in the Excel. | p8 |
|  | 13d | Describe any methods used to synthesize results and provide a rationale for the choice(s). If meta-analysis was performed, describe the model(s), method(s) to identify the presence and extent of statistical heterogeneity, and software package(s) used.  Our research was not related to this problem. | Not applicable |
|  | 13e | Describe any methods used to explore possible causes of heterogeneity among study results (e.g. subgroup analysis, meta-regression).  Our research was not related to this problem. | Not applicable |
|  | 13f | Describe any sensitivity analyses conducted to assess robustness of the synthesized results.  Our research was not related to this problem. | Not applicable |
| Reporting bias assessment | 14 | Describe any methods used to assess risk of bias due to missing results in a synthesis (arising from reporting biases).  Our research was not related to this problem. We do not have any missing results. | Not applicable |
| Certainty assessment | 15 | Describe any methods used to assess certainty (or confidence) in the body of evidence for an outcome.  We did not explicitly perform any assessments using standardized criteria or tools for the studies included. This decision was driven by the primary goal of our study, which was to comprehensively gather relevant papers. However, it's worth noting that we did encounter certain papers from Chinese databases that included non-academic content, such as the article with the title '20 New Concepts of Healthy Living.' Nevertheless, we did take the quality of these studies from Chinese databases into account during our analysis. This consideration involved a qualitative assessment that considered factors such as study design, methodology, and the presence of bias or limitations. These factors were factored into our interpretation of the results. | Not applicable |
| **RESULTS** | | |  |
| Study selection | 16a | Describe the results of the search and selection process, from the number of records identified in the search to the number of studies included in the review, ideally using a flow diagram.  See flow chart 1 and 2. | p27 |
|  | 16b | Cite studies that might appear to meet the inclusion criteria, but which were excluded, and explain why they were excluded.  It also shown in the flow chart 1 and 2, where we have listed the exclusion reasons along with the respective count of excluded papers for each criterion. | p27 |
| Study characteristics | 17 | Cite each included study and present its characteristics.  Appendix 2 is the data extraction table, including the title, author, publish year, region, study aim, methods, and results of all eligible papers. | Appendix 2 |
| Risk of bias in studies | 18 | Present assessments of risk of bias for each included study.  Our research was not related to this problem. | Not applicable |
| Results of individual studies | 19 | For all outcomes, present, for each study: (a) summary statistics for each group (where appropriate) and (b) an effect estimate and its precision (e.g. confidence/credible interval), ideally using structured tables or plots.  Figure 3 and Figure 4 are the results we summarized into frameworks. | p28 |
| Results of syntheses | 20a | For each synthesis, briefly summarise the characteristics and risk of bias among contributing studies.  Appendix 2 is the data extraction table, including the title, author, publish year, region, study aim, methods, and results of all eligible papers without the risk of bias, which is not related to our study. | Appendix 2 |
|  | 20b | Present results of all statistical syntheses conducted. If meta-analysis was done, present for each the summary estimate and its precision (e.g. confidence/credible interval) and measures of statistical heterogeneity. If comparing groups, describe the direction of the effect.  Our research was not related to this problem. | Not applicable |
|  | 20c | Present results of all investigations of possible causes of heterogeneity among study results.  Our research was not related to this problem. | Not applicable |
|  | 20d | Present results of all sensitivity analyses conducted to assess the robustness of the synthesized results.  Our research was not related to this problem. | Not applicable |
| Reporting biases | 21 | Present assessments of risk of bias due to missing results (arising from reporting biases) for each synthesis assessed.  Our research was not related to this problem. | Not applicable |
| Certainty of evidence | 22 | Present assessments of certainty (or confidence) in the body of evidence for each outcome assessed.  Our research was not related to this problem. | Not applicable |
| **DISCUSSION** | | |  |
| Discussion | 23a | Provide a general interpretation of the results in the context of other evidence. | p15 |
|  | 23b | Discuss any limitations of the evidence included in the review. | p19 |
|  | 23c | Discuss any limitations of the review processes used. | p19 |
|  | 23d | Discuss implications of the results for practice, policy, and future research. | p18-p19 |
| **OTHER INFORMATION** | | |  |
| Registration and protocol | 24a | Provide registration information for the review, including register name and registration number, or state that the review was not registered.  The review was not registered. | Not applicable |
|  | 24b | Indicate where the review protocol can be accessed, or state that a protocol was not prepared.  A protocol was not prepared. | Not applicable |
|  | 24c | Describe and explain any amendments to information provided at registration or in the protocol.  Our research was not related to this problem. | Not applicable |
| Support | 25 | Describe sources of financial or non-financial support for the review, and the role of the funders or sponsors in the review.  Open Access funding enabled and organized by Erasmus Medical Center. This work was funded by EuroQol Foundation, the Netherlands. | p20 |
| Competing interests | 26 | Declare any competing interests of review authors.  The authors have no relevant financial or non-financial interests to disclose. | p20 |
| Availability of data, code and other materials | 27 | Report which of the following are publicly available and where they can be found: template data collection forms; data extracted from included studies; data used for all analyses; analytic code; any other materials used in the review. | p20 |

*From:*  Page MJ, McKenzie JE, Bossuyt PM, Boutron I, Hoffmann TC, Mulrow CD, et al. The PRISMA 2020 statement: an updated guideline for reporting systematic reviews. BMJ 2021;372:n71. doi: 10.1136/bmj.n71

For more information, visit: <http://www.prisma-statement.org/>
