# Supplementary material for: Differences and common ground in the frameworks of health-related quality of life in traditional Chinese medicine and modern medicine: a systematic review
Source: Qual Life Res. 2024 May 13;33(7):1795–806. doi: 10.1007/s11136-024-03669-1 (PMC11176225; doi:10.1007/s11136-024-03669-1)
Supplement: Supplementary file 7 — Supplementary file7 (DOCX 19 KB) [file 11136_2024_3669_MOESM7_ESM.docx]

**Appendix 7**

In this appendix, we have compiled a list of all the facets from both Figure 3 and Figure 4, along with specific references for each facet.

- **Facets from Traditional Chinese Medicine (TCM) framework:**

**6 energy**：

- Leung, K. F., Liu, F. B., Zhao, L., Fang, J. Q., Chan, K., & Lin, L. Z. (2005). Development and validation of the Chinese Quality of Life Instrument. Health Qual Life Outcomes, 3, 26.[17]
- Li X.(2007). Study and Evaluation of PRO（patient-reported outcomes） Properties of the Chinese Health-Related Quality of Life Scale. Unpublished,Guang zhou zhong yi yao da xue.[25]
- Lang J. (2005). Development, Evaluation, and Initial Application of the Chinese Health-Related Quality of Life Scale (CH-QOL). Unpublished, Zhong shan da xue.[37]
- Liu F., Zhao L, Liang G., Lin L.(2007). Theoretical and Structural Model Exploration of Traditional Chinese Medicine Health Status Scale. Xin zhong yao(09), 10-12. [28]
- Wu D., Lai S.(2007). Discussion on the Concept of Health in Traditional Chinese Medicine and its Operationalization in Measurement. Zhong guo zhong xi yi jie he za zhi(02), 174-177.[38]
- Zhu W., Gao H., Zhang M., Shi L., Tao L., Chen Y., Shi L., Yu C., Han S, Li S.,Wei J. (2022). The development of the Traditional Chinese Medicine Quality of Life Assessment Scale (CQ-11D). Zhong guo yao wu jing ji xue, 17(05), 10-15+39.[33]

**5 sleep**

- Lang J. (2005). Development, Evaluation, and Initial Application of the Chinese Health-Related Quality of Life Scale (CH-QOL). Unpublished, Zhong shan da xue.[37]
- Li X.(2007). Study and Evaluation of PRO（patient-reported outcomes） Properties of the Chinese Health-Related Quality of Life Scale. Unpublished,Guang zhou zhong yi yao da xue.[25]
- Lang J. (2005). Development, Evaluation, and Initial Application of the Chinese Health-Related Quality of Life Scale (CH-QOL). Unpublished, Zhong shan da xue.[37]
- Wu D., Lai S.(2007). Discussion on the Concept of Health in Traditional Chinese Medicine and its Operationalization in Measurement. Zhong guo zhong xi yi jie he za zhi(02), 174-177.[38]
- Zhu W., Gao H., Zhang M., Shi L., Tao L., Chen Y., Shi L., Yu C., Han S, Li S.,Wei J. (2022). The development of the Traditional Chinese Medicine Quality of Life Assessment Scale (CQ-11D). Zhong guo yao wu jing ji xue, 17(05), 10-15+39.[33]

**5 appetite&digestion**

- Lang J. (2005). Development, Evaluation, and Initial Application of the Chinese Health-Related Quality of Life Scale (CH-QOL). Unpublished, Zhong shan da xue.[37]
- Li X.(2007). Study and Evaluation of PRO（patient-reported outcomes） Properties of the Chinese Health-Related Quality of Life Scale. Unpublished,Guang zhou zhong yi yao da xue.[25]
- Leung, K. F., Liu, F. B., Zhao, L., Fang, J. Q., Chan, K., & Lin, L. Z. (2005). Development and validation of the Chinese Quality of Life Instrument. Health Qual Life Outcomes, 3, 26.[17]
- Liu F., Zhao L, Liang G., Lin L.(2007). Theoretical and Structural Model Exploration of Traditional Chinese Medicine Health Status Scale. Xin zhong yao(09), 10-12. [28]
- Zhu W., Gao H., Zhang M., Shi L., Tao L., Chen Y., Shi L., Yu C., Han S, Li S.,Wei J. (2022). The development of the Traditional Chinese Medicine Quality of Life Assessment Scale (CQ-11D). Zhong guo yao wu jing ji xue, 17(05), 10-15+39.[33]

**3 complexion**

- Lang J. (2005). Development, Evaluation, and Initial Application of the Chinese Health-Related Quality of Life Scale (CH-QOL). Unpublished, Zhong shan da xue.[37]
- Li X.(2007). Study and Evaluation of PRO（patient-reported outcomes） Properties of the Chinese Health-Related Quality of Life Scale. Unpublished,Guang zhou zhong yi yao da xue.[25]
- Leung, K. F., Liu, F. B., Zhao, L., Fang, J. Q., Chan, K., & Lin, L. Z. (2005). Development and validation of the Chinese Quality of Life Instrument. Health Qual Life Outcomes, 3, 26.[17]

**5 stool**

- Li X.(2007). Study and Evaluation of PRO（patient-reported outcomes） Properties of the Chinese Health-Related Quality of Life Scale. Unpublished,Guang zhou zhong yi yao da xue.[25]
- Lang J. (2005). Development, Evaluation, and Initial Application of the Chinese Health-Related Quality of Life Scale (CH-QOL). Unpublished, Zhong shan da xue.[37]
- Wu D., Lai S.(2007). Discussion on the Concept of Health in Traditional Chinese Medicine and its Operationalization in Measurement. Zhong guo zhong xi yi jie he za zhi(02), 174-177.[38]
- Zhu W., Gao H., Zhang M., Shi L., Tao L., Chen Y., Shi L., Yu C., Han S, Li S.,Wei J. (2022). The development of the Traditional Chinese Medicine Quality of Life Assessment Scale (CQ-11D). Zhong guo yao wu jing ji xue, 17(05), 10-15+39.[33]
- Liu F., Zhao L, Liang G., Lin L.(2007). Theoretical and Structural Model Exploration of Traditional Chinese Medicine Health Status Scale. Xin zhong yao(09), 10-12. [28]

**3 mobility**

- Li X.(2007). Study and Evaluation of PRO（patient-reported outcomes） Properties of the Chinese Health-Related Quality of Life Scale. Unpublished,Guang zhou zhong yi yao da xue.[25]
- Lang J. (2005). Development, Evaluation, and Initial Application of the Chinese Health-Related Quality of Life Scale (CH-QOL). Unpublished, Zhong shan da xue.[37]
- Zhu W., Gao H., Zhang M., Shi L., Tao L., Chen Y., Shi L., Yu C., Han S, Li S.,Wei J. (2022). The development of the Traditional Chinese Medicine Quality of Life Assessment Scale (CQ-11D). Zhong guo yao wu jing ji xue, 17(05), 10-15+39.[33]

**1 self-care**

- Zhu W., Gao H., Zhang M., Shi L., Tao L., Chen Y., Shi L., Yu C., Han S, Li S.,Wei J. (2022). The development of the Traditional Chinese Medicine Quality of Life Assessment Scale (CQ-11D). Zhong guo yao wu jing ji xue, 17(05), 10-15+39.[33]

**3 discomfort**

- Zhu W., Gao H., Zhang M., Shi L., Tao L., Chen Y., Shi L., Yu C., Han S, Li S.,Wei J. (2022). The development of the Traditional Chinese Medicine Quality of Life Assessment Scale (CQ-11D). Zhong guo yao wu jing ji xue, 17(05), 10-15+39.[33]
- Li X.(2007). Study and Evaluation of PRO（patient-reported outcomes） Properties of the Chinese Health-Related Quality of Life Scale. Unpublished,Guang zhou zhong yi yao da xue.[25]
- Lang J. (2005). Development, Evaluation, and Initial Application of the Chinese Health-Related Quality of Life Scale (CH-QOL). Unpublished, Zhong shan da xue.[37]

**2 pain**

- Liu F., Zhao L, Liang G., Lin L.(2007). Theoretical and Structural Model Exploration of Traditional Chinese Medicine Health Status Scale. Xin zhong yao(09), 10-12. [28]
- Zhu W., Gao H., Zhang M., Shi L., Tao L., Chen Y., Shi L., Yu C., Han S, Li S.,Wei J. (2022). The development of the Traditional Chinese Medicine Quality of Life Assessment Scale (CQ-11D). Zhong guo yao wu jing ji xue, 17(05), 10-15+39.[33]

**3 urinate**

- Li X.(2007). Study and Evaluation of PRO（patient-reported outcomes） Properties of the Chinese Health-Related Quality of Life Scale. Unpublished,Guang zhou zhong yi yao da xue.[25]
- Liu F., Zhao L, Liang G., Lin L.(2007). Theoretical and Structural Model Exploration of Traditional Chinese Medicine Health Status Scale. Xin zhong yao(09), 10-12. [28]
- Wu D., Lai S.(2007). Discussion on the Concept of Health in Traditional Chinese Medicine and its Operationalization in Measurement. Zhong guo zhong xi yi jie he za zhi(02), 174-177.[38]

**2 sound**

- Wu D., Lai S.(2007). Discussion on the Concept of Health in Traditional Chinese Medicine and its Operationalization in Measurement. Zhong guo zhong xi yi jie he za zhi(02), 174-177.[38]
- Li X.(2007). Study and Evaluation of PRO（patient-reported outcomes） Properties of the Chinese Health-Related Quality of Life Scale. Unpublished,Guang zhou zhong yi yao da xue.[25]

**1 constitution**

- Liu F., Zhao L, Liang G., Lin L.(2007). Theoretical and Structural Model Exploration of Traditional Chinese Medicine Health Status Scale. Xin zhong yao(09), 10-12. [28]

**4 thinking**

- Leung, K. F., Liu, F. B., Zhao, L., Fang, J. Q., Chan, K., & Lin, L. Z. (2005). Development and validation of the Chinese Quality of Life Instrument. Health Qual Life Outcomes, 3, 26.[17]
- Li X.(2007). Study and Evaluation of PRO（patient-reported outcomes） Properties of the Chinese Health-Related Quality of Life Scale. Unpublished,Guang zhou zhong yi yao da xue.[25]
- Lang J. (2005). Development, Evaluation, and Initial Application of the Chinese Health-Related Quality of Life Scale (CH-QOL). Unpublished, Zhong shan da xue.[37]
- Wu D., Lai S.(2007). Discussion on the Concept of Health in Traditional Chinese Medicine and its Operationalization in Measurement. Zhong guo zhong xi yi jie he za zhi(02), 174-177.[38]

**3 verbal expression**

- Leung, K. F., Liu, F. B., Zhao, L., Fang, J. Q., Chan, K., & Lin, L. Z. (2005). Development and validation of the Chinese Quality of Life Instrument. Health Qual Life Outcomes, 3, 26.[17]
- Li X.(2007). Study and Evaluation of PRO（patient-reported outcomes） Properties of the Chinese Health-Related Quality of Life Scale. Unpublished,Guang zhou zhong yi yao da xue.[25]
- Lang J. (2005). Development, Evaluation, and Initial Application of the Chinese Health-Related Quality of Life Scale (CH-QOL). Unpublished, Zhong shan da xue.[37]

**3 consciousness**

- Leung, K. F., Liu, F. B., Zhao, L., Fang, J. Q., Chan, K., & Lin, L. Z. (2005). Development and validation of the Chinese Quality of Life Instrument. Health Qual Life Outcomes, 3, 26.[17]
- Li X.(2007). Study and Evaluation of PRO（patient-reported outcomes） Properties of the Chinese Health-Related Quality of Life Scale. Unpublished,Guang zhou zhong yi yao da xue.[25]
- Lang J. (2005). Development, Evaluation, and Initial Application of the Chinese Health-Related Quality of Life Scale (CH-QOL). Unpublished, Zhong shan da xue.[37]

**2 spirit of eye**

- Leung, K. F., Liu, F. B., Zhao, L., Fang, J. Q., Chan, K., & Lin, L. Z. (2005). Development and validation of the Chinese Quality of Life Instrument. Health Qual Life Outcomes, 3, 26.[17]
- Li X.(2007). Study and Evaluation of PRO（patient-reported outcomes） Properties of the Chinese Health-Related Quality of Life Scale. Unpublished,Guang zhou zhong yi yao da xue.[25]

**2 memory**

- Li X.(2007). Study and Evaluation of PRO（patient-reported outcomes） Properties of the Chinese Health-Related Quality of Life Scale. Unpublished,Guang zhou zhong yi yao da xue.[25]
- Lang J. (2005). Development, Evaluation, and Initial Application of the Chinese Health-Related Quality of Life Scale (CH-QOL). Unpublished, Zhong shan da xue.[37]

**2 concentration**

- Li X.(2007). Study and Evaluation of PRO（patient-reported outcomes） Properties of the Chinese Health-Related Quality of Life Scale. Unpublished,Guang zhou zhong yi yao da xue.[25]
- Lang J. (2005). Development, Evaluation, and Initial Application of the Chinese Health-Related Quality of Life Scale (CH-QOL). Unpublished, Zhong shan da xue.[37]

**3 fatigue**

- Li X.(2007). Study and Evaluation of PRO（patient-reported outcomes） Properties of the Chinese Health-Related Quality of Life Scale. Unpublished,Guang zhou zhong yi yao da xue.[25]
- Lang J. (2005). Development, Evaluation, and Initial Application of the Chinese Health-Related Quality of Life Scale (CH-QOL). Unpublished, Zhong shan da xue.[37]
- Zhu W., Gao H., Zhang M., Shi L., Tao L., Chen Y., Shi L., Yu C., Han S, Li S.,Wei J. (2022). The development of the Traditional Chinese Medicine Quality of Life Assessment Scale (CQ-11D). Zhong guo yao wu jing ji xue, 17(05), 10-15+39.[33]

**1 confidence**

- Wu D., Lai S.(2007). Discussion on the Concept of Health in Traditional Chinese Medicine and its Operationalization in Measurement. Zhong guo zhong xi yi jie he za zhi(02), 174-177.[38]

**1 satisfaction**

- Wu D., Lai S.(2007). Discussion on the Concept of Health in Traditional Chinese Medicine and its Operationalization in Measurement. Zhong guo zhong xi yi jie he za zhi(02), 174-177.[38]

**4 climate adaptation and adjustment**

- Leung, K. F., Liu, F. B., Zhao, L., Fang, J. Q., Chan, K., & Lin, L. Z. (2005). Development and validation of the Chinese Quality of Life Instrument. Health Qual Life Outcomes, 3, 26.[17]
- Li X.(2007). Study and Evaluation of PRO（patient-reported outcomes） Properties of the Chinese Health-Related Quality of Life Scale. Unpublished,Guang zhou zhong yi yao da xue.[25]
- Lang J. (2005). Development, Evaluation, and Initial Application of the Chinese Health-Related Quality of Life Scale (CH-QOL). Unpublished, Zhong shan da xue.[37]
- Wu D., Lai S.(2007). Discussion on the Concept of Health in Traditional Chinese Medicine and its Operationalization in Measurement. Zhong guo zhong xi yi jie he za zhi(02), 174-177.[38]

**2 dwelling conditions**

- Lang J. (2005). Development, Evaluation, and Initial Application of the Chinese Health-Related Quality of Life Scale (CH-QOL). Unpublished, Zhong shan da xue.[37]
- Wu D., Lai S.(2007). Discussion on the Concept of Health in Traditional Chinese Medicine and its Operationalization in Measurement. Zhong guo zhong xi yi jie he za zhi(02), 174-177.[38]

**3 socialization(e.g. feeling lonely, sex life, communication)**

- Li X.(2007). Study and Evaluation of PRO（patient-reported outcomes） Properties of the Chinese Health-Related Quality of Life Scale. Unpublished,Guang zhou zhong yi yao da xue.[25]
- Lang J. (2005). Development, Evaluation, and Initial Application of the Chinese Health-Related Quality of Life Scale (CH-QOL). Unpublished, Zhong shan da xue.[37]
- Wu D., Lai S.(2007). Discussion on the Concept of Health in Traditional Chinese Medicine and its Operationalization in Measurement. Zhong guo zhong xi yi jie he za zhi(02), 174-177.[38]

**2 family(e.g. relationships, support, fights)**

- Li X.(2007). Study and Evaluation of PRO（patient-reported outcomes） Properties of the Chinese Health-Related Quality of Life Scale. Unpublished,Guang zhou zhong yi yao da xue.[25]
- Lang J. (2005). Development, Evaluation, and Initial Application of the Chinese Health-Related Quality of Life Scale (CH-QOL). Unpublished, Zhong shan da xue.[37]

**2 work(e.g. relationships, performance)**

- Li X.(2007). Study and Evaluation of PRO（patient-reported outcomes） Properties of the Chinese Health-Related Quality of Life Scale. Unpublished,Guang zhou zhong yi yao da xue.[25]
- Lang J. (2005). Development, Evaluation, and Initial Application of the Chinese Health-Related Quality of Life Scale (CH-QOL). Unpublished, Zhong shan da xue.[37]

**2 economic conditions**

- Li X.(2007). Study and Evaluation of PRO（patient-reported outcomes） Properties of the Chinese Health-Related Quality of Life Scale. Unpublished,Guang zhou zhong yi yao da xue.[25]
- Lang J. (2005). Development, Evaluation, and Initial Application of the Chinese Health-Related Quality of Life Scale (CH-QOL). Unpublished, Zhong shan da xue.[37]

**4 anger**

- Leung, K. F., Liu, F. B., Zhao, L., Fang, J. Q., Chan, K., & Lin, L. Z. (2005). Development and validation of the Chinese Quality of Life Instrument. Health Qual Life Outcomes, 3, 26.[17]
- Li X.(2007). Study and Evaluation of PRO（patient-reported outcomes） Properties of the Chinese Health-Related Quality of Life Scale. Unpublished,Guang zhou zhong yi yao da xue.[25]
- Lang J. (2005). Development, Evaluation, and Initial Application of the Chinese Health-Related Quality of Life Scale (CH-QOL). Unpublished, Zhong shan da xue.[37]
- Zhu W., Gao H., Zhang M., Shi L., Tao L., Chen Y., Shi L., Yu C., Han S, Li S.,Wei J. (2022). The development of the Traditional Chinese Medicine Quality of Life Assessment Scale (CQ-11D). Zhong guo yao wu jing ji xue, 17(05), 10-15+39.[33]

**3 joy**

- Leung, K. F., Liu, F. B., Zhao, L., Fang, J. Q., Chan, K., & Lin, L. Z. (2005). Development and validation of the Chinese Quality of Life Instrument. Health Qual Life Outcomes, 3, 26.[17]
- Li X.(2007). Study and Evaluation of PRO（patient-reported outcomes） Properties of the Chinese Health-Related Quality of Life Scale. Unpublished,Guang zhou zhong yi yao da xue.[25]
- Lang J. (2005). Development, Evaluation, and Initial Application of the Chinese Health-Related Quality of Life Scale (CH-QOL). Unpublished, Zhong shan da xue.[37]

**2 grief**

- Li X.(2007). Study and Evaluation of PRO（patient-reported outcomes） Properties of the Chinese Health-Related Quality of Life Scale. Unpublished,Guang zhou zhong yi yao da xue.[25]
- Lang J. (2005). Development, Evaluation, and Initial Application of the Chinese Health-Related Quality of Life Scale (CH-QOL). Unpublished, Zhong shan da xue.[37]

**2 worry**

- Li X.(2007). Study and Evaluation of PRO（patient-reported outcomes） Properties of the Chinese Health-Related Quality of Life Scale. Unpublished,Guang zhou zhong yi yao da xue.[25]
- Lang J. (2005). Development, Evaluation, and Initial Application of the Chinese Health-Related Quality of Life Scale (CH-QOL). Unpublished, Zhong shan da xue.[37]

**2 fear**

- Leung, K. F., Liu, F. B., Zhao, L., Fang, J. Q., Chan, K., & Lin, L. Z. (2005). Development and validation of the Chinese Quality of Life Instrument. Health Qual Life Outcomes, 3, 26.[17]
- Lang J. (2005). Development, Evaluation, and Initial Application of the Chinese Health-Related Quality of Life Scale (CH-QOL). Unpublished, Zhong shan da xue.[37]

**1 pensiveness**

- Lang J. (2005). Development, Evaluation, and Initial Application of the Chinese Health-Related Quality of Life Scale (CH-QOL). Unpublished, Zhong shan da xue.[37]

**3 anxiety**

- Li X.(2007). Study and Evaluation of PRO（patient-reported outcomes） Properties of the Chinese Health-Related Quality of Life Scale. Unpublished,Guang zhou zhong yi yao da xue.[25]
- Lang J. (2005). Development, Evaluation, and Initial Application of the Chinese Health-Related Quality of Life Scale (CH-QOL). Unpublished, Zhong shan da xue.[37]
- Zhu W., Gao H., Zhang M., Shi L., Tao L., Chen Y., Shi L., Yu C., Han S, Li S.,Wei J. (2022). The development of the Traditional Chinese Medicine Quality of Life Assessment Scale (CQ-11D). Zhong guo yao wu jing ji xue, 17(05), 10-15+39.[33]

**Facets from Modern Medicine (MM) framework:**

**5 appetite**

- Wu J, He X, Chen P, et al. China Health Related Outcomes Measures (CHROME): Development of a New Generic Preference-Based Measure for the Chinese Population[J]. PharmacoEconomics, 2022, 40(10): 957-969.
- Mao Z, Ahmed S, Graham C, et al. The unfolding method to explore health-related quality of life constructs in a Chinese general population[J]. Value in Health, 2021, 24(6): 846-854T
- Li, M., Bao, Z., Lv, G., Zhou, J., Chen, P., & Luo, N. (2021). Culture-Related Health Disparities in Quality of Life: Assessment of Instrument Dimensions Among Chinese. Front Public Health, 9, 663904.[10]
- Wu Y., Xie G., Li Y, Zhou B., Zhang P., Shi P, Ren F., Ma L.(2005). Development and Evaluation of a General Quality of Life Scale for Chinese People. Zhong hua liu xing bing xue za zhi(10), 27-32.[23]
- Mao, Z., Ahmed, S., Graham, C., & Kind, P. (2020). Exploring subjective constructions of health in China: a Q-methodological investigation. Health Qual Life Outcomes, 18(1), 165.[11]

**5 sleep**

- Wu J, He X, Chen P, et al. China Health Related Outcomes Measures (CHROME): Development of a New Generic Preference-Based Measure for the Chinese Population[J]. PharmacoEconomics, 2022, 40(10): 957-969.
- Mao Z, Ahmed S, Graham C, et al. The unfolding method to explore health-related quality of life constructs in a Chinese general population[J]. Value in Health, 2021, 24(6): 846-854
- Li, M., Bao, Z., Lv, G., Zhou, J., Chen, P., & Luo, N. (2021). Culture-Related Health Disparities in Quality of Life: Assessment of Instrument Dimensions Among Chinese. Front Public Health, 9, 663904.[10]
- Wu Y., Xie G., Li Y, Zhou B., Zhang P., Shi P, Ren F., Ma L.(2005). Development and Evaluation of a General Quality of Life Scale for Chinese People. Zhong hua liu xing bing xue za zhi(10), 27-32.[23]
- Mao, Z., Ahmed, S., Graham, C., & Kind, P. (2020). Exploring subjective constructions of health in China: a Q-methodological investigation. Health Qual Life Outcomes, 18(1), 165.[11]

**6 mobility**

- Shiroiwa T, Murata T, Ahn J, et al. Developing a New Region-Specific Preference-Based Measure in East and Southeast Asia[J]. Value in health regional issues, 2022, 32: 62-69
- Wu J, He X, Chen P, et al. China Health Related Outcomes Measures (CHROME): Development of a New Generic Preference-Based Measure for the Chinese Population[J]. PharmacoEconomics, 2022, 40(10): 957-969.
- Mao Z, Ahmed S, Graham C, et al. The unfolding method to explore health-related quality of life constructs in a Chinese general population[J]. Value in Health, 2021, 24(6): 846-854
- Li, M., Bao, Z., Lv, G., Zhou, J., Chen, P., & Luo, N. (2021). Culture-Related Health Disparities in Quality of Life: Assessment of Instrument Dimensions Among Chinese. Front Public Health, 9, 663904.[10]
- Wu Y., Xie G., Li Y, Zhou B., Zhang P., Shi P, Ren F., Ma L.(2005). Development and Evaluation of a General Quality of Life Scale for Chinese People. Zhong hua liu xing bing xue za zhi(10), 27-32.[23]
- Mao, Z., Ahmed, S., Graham, C., & Kind, P. (2020). Exploring subjective constructions of health in China: a Q-methodological investigation. Health Qual Life Outcomes, 18(1), 165.[11]

**5 daily activities**

- Wu J, He X, Chen P, et al. China Health Related Outcomes Measures (CHROME): Development of a New Generic Preference-Based Measure for the Chinese Population[J]. PharmacoEconomics, 2022, 40(10): 957-969.
- Mao Z, Ahmed S, Graham C, et al. The unfolding method to explore health-related quality of life constructs in a Chinese general population[J]. Value in Health, 2021, 24(6): 846-854
- Li, M., Bao, Z., Lv, G., Zhou, J., Chen, P., & Luo, N. (2021). Culture-Related Health Disparities in Quality of Life: Assessment of Instrument Dimensions Among Chinese. Front Public Health, 9, 663904.[10]
- Wu Y., Xie G., Li Y, Zhou B., Zhang P., Shi P, Ren F., Ma L.(2005). Development and Evaluation of a General Quality of Life Scale for Chinese People. Zhong hua liu xing bing xue za zhi(10), 27-32.[23]
- Mao, Z., Ahmed, S., Graham, C., & Kind, P. (2020). Exploring subjective constructions of health in China: a Q-methodological investigation. Health Qual Life Outcomes, 18(1), 165.[11]

**4 self-care**

- Mao Z, Ahmed S, Graham C, et al. The unfolding method to explore health-related quality of life constructs in a Chinese general population[J]. Value in Health, 2021, 24(6): 846-854
- Li, M., Bao, Z., Lv, G., Zhou, J., Chen, P., & Luo, N. (2021). Culture-Related Health Disparities in Quality of Life: Assessment of Instrument Dimensions Among Chinese. Front Public Health, 9, 663904.[10]
- Wu Y., Xie G., Li Y, Zhou B., Zhang P., Shi P, Ren F., Ma L.(2005). Development and Evaluation of a General Quality of Life Scale for Chinese People. Zhong hua liu xing bing xue za zhi(10), 27-32.[23]
- Mao, Z., Ahmed, S., Graham, C., & Kind, P. (2020). Exploring subjective constructions of health in China: a Q-methodological investigation. Health Qual Life Outcomes, 18(1), 165.[11]

**3 vision**

- Wu J, He X, Chen P, et al. China Health Related Outcomes Measures (CHROME): Development of a New Generic Preference-Based Measure for the Chinese Population[J]. PharmacoEconomics, 2022, 40(10): 957-969.
- Mao Z, Ahmed S, Graham C, et al. The unfolding method to explore health-related quality of life constructs in a Chinese general population[J]. Value in Health, 2021, 24(6): 846-854
- Mao, Z., Ahmed, S., Graham, C., & Kind, P. (2020). Exploring subjective constructions of health in China: a Q-methodological investigation. Health Qual Life Outcomes, 18(1), 165.[11]

**3 hearing**

- Wu J, He X, Chen P, et al. China Health Related Outcomes Measures (CHROME): Development of a New Generic Preference-Based Measure for the Chinese Population[J]. PharmacoEconomics, 2022, 40(10): 957-969.
- Mao Z, Ahmed S, Graham C, et al. The unfolding method to explore health-related quality of life constructs in a Chinese general population[J]. Value in Health, 2021, 24(6): 846-854
- Mao, Z., Ahmed, S., Graham, C., & Kind, P. (2020). Exploring subjective constructions of health in China: a Q-methodological investigation. Health Qual Life Outcomes, 18(1), 165.[11]

**2 body constitution**

- Mao Z, Ahmed S, Graham C, et al. The unfolding method to explore health-related quality of life constructs in a Chinese general population[J]. Value in Health, 2021, 24(6): 846-854
- Mao, Z., Ahmed, S., Graham, C., & Kind, P. (2020). Exploring subjective constructions of health in China: a Q-methodological investigation. Health Qual Life Outcomes, 18(1), 165.[11]

**1 physical exercise and play**

- Li, M., Bao, Z., Lv, G., Zhou, J., Chen, P., & Luo, N. (2021). Culture-Related Health Disparities in Quality of Life: Assessment of Instrument Dimensions Among Chinese. Front Public Health, 9, 663904.[10]

**2 heavy physical work**

- Li, M., Bao, Z., Lv, G., Zhou, J., Chen, P., & Luo, N. (2021). Culture-Related Health Disparities in Quality of Life: Assessment of Instrument Dimensions Among Chinese. Front Public Health, 9, 663904.[10]
- Wu Y., Xie G., Li Y, Zhou B., Zhang P., Shi P, Ren F., Ma L.(2005). Development and Evaluation of a General Quality of Life Scale for Chinese People. Zhong hua liu xing bing xue za zhi(10), 27-32.[23]

**6 pain**

- Shiroiwa T, Murata T, Ahn J, et al. Developing a New Region-Specific Preference-Based Measure in East and Southeast Asia[J]. Value in health regional issues, 2022, 32: 62-69
- Wu J, He X, Chen P, et al. China Health Related Outcomes Measures (CHROME): Development of a New Generic Preference-Based Measure for the Chinese Population[J]. PharmacoEconomics, 2022, 40(10): 957-969.
- Mao Z, Ahmed S, Graham C, et al. The unfolding method to explore health-related quality of life constructs in a Chinese general population[J]. Value in Health, 2021, 24(6): 846-854
- Li, M., Bao, Z., Lv, G., Zhou, J., Chen, P., & Luo, N. (2021). Culture-Related Health Disparities in Quality of Life: Assessment of Instrument Dimensions Among Chinese. Front Public Health, 9, 663904.[10]
- Wu Y., Xie G., Li Y, Zhou B., Zhang P., Shi P, Ren F., Ma L.(2005). Development and Evaluation of a General Quality of Life Scale for Chinese People. Zhong hua liu xing bing xue za zhi(10), 27-32.[23]
- Mao, Z., Ahmed, S., Graham, C., & Kind, P. (2020). Exploring subjective constructions of health in China: a Q-methodological investigation. Health Qual Life Outcomes, 18(1), 165.[11]

**4 fatigue**

- Wu J, He X, Chen P, et al. China Health Related Outcomes Measures (CHROME): Development of a New Generic Preference-Based Measure for the Chinese Population[J]. PharmacoEconomics, 2022, 40(10): 957-969.
- Mao Z, Ahmed S, Graham C, et al. The unfolding method to explore health-related quality of life constructs in a Chinese general population[J]. Value in Health, 2021, 24(6): 846-854
- Wu Y., Xie G., Li Y, Zhou B., Zhang P., Shi P, Ren F., Ma L.(2005). Development and Evaluation of a General Quality of Life Scale for Chinese People. Zhong hua liu xing bing xue za zhi(10), 27-32.[23]
- Mao, Z., Ahmed, S., Graham, C., & Kind, P. (2020). Exploring subjective constructions of health in China: a Q-methodological investigation. Health Qual Life Outcomes, 18(1), 165.[11]

**4 energy**

- Shiroiwa T, Murata T, Ahn J, et al. Developing a New Region-Specific Preference-Based Measure in East and Southeast Asia[J]. Value in health regional issues, 2022, 32: 62-69
- Li, M., Bao, Z., Lv, G., Zhou, J., Chen, P., & Luo, N. (2021). Culture-Related Health Disparities in Quality of Life: Assessment of Instrument Dimensions Among Chinese. Front Public Health, 9, 663904.[10]
- Mao Z, Ahmed S, Graham C, et al. The unfolding method to explore health-related quality of life constructs in a Chinese general population[J]. Value in Health, 2021, 24(6): 846-854
- Mao, Z., Ahmed, S., Graham, C., & Kind, P. (2020). Exploring subjective constructions of health in China: a Q-methodological investigation. Health Qual Life Outcomes, 18(1), 165.[11]

**1 body weight**

- Mao Z, Ahmed S, Graham C, et al. The unfolding method to explore health-related quality of life constructs in a Chinese general population[J]. Value in Health, 2021, 24(6): 846-854

**2 dependence on medication**

- Mao Z, Ahmed S, Graham C, et al. The unfolding method to explore health-related quality of life constructs in a Chinese general population[J]. Value in Health, 2021, 24(6): 846-854
- Wu Y., Xie G., Li Y, Zhou B., Zhang P., Shi P, Ren F., Ma L.(2005). Development and Evaluation of a General Quality of Life Scale for Chinese People. Zhong hua liu xing bing xue za zhi(10), 27-32.[23]

**2 discomfort**

- Mao Z, Ahmed S, Graham C, et al. The unfolding method to explore health-related quality of life constructs in a Chinese general population[J]. Value in Health, 2021, 24(6): 846-854
- Mao, Z., Ahmed, S., Graham, C., & Kind, P. (2020). Exploring subjective constructions of health in China: a Q-methodological investigation. Health Qual Life Outcomes, 18(1), 165.[11]

**1 color of face**

- Mao Z, Ahmed S, Graham C, et al. The unfolding method to explore health-related quality of life constructs in a Chinese general population[J]. Value in Health, 2021, 24(6): 846-854

**3 body strength**

- Mao Z, Ahmed S, Graham C, et al. The unfolding method to explore health-related quality of life constructs in a Chinese general population[J]. Value in Health, 2021, 24(6): 846-854
- Li, M., Bao, Z., Lv, G., Zhou, J., Chen, P., & Luo, N. (2021). Culture-Related Health Disparities in Quality of Life: Assessment of Instrument Dimensions Among Chinese. Front Public Health, 9, 663904.[10]
- Mao, Z., Ahmed, S., Graham, C., & Kind, P. (2020). Exploring subjective constructions of health in China: a Q-methodological investigation. Health Qual Life Outcomes, 18(1), 165.[11]

**1 family medical history**

- Mao Z, Ahmed S, Graham C, et al. The unfolding method to explore health-related quality of life constructs in a Chinese general population[J]. Value in Health, 2021, 24(6): 846-854

**4 depression**

- Wu J, He X, Chen P, et al. China Health Related Outcomes Measures (CHROME): Development of a New Generic Preference-Based Measure for the Chinese Population[J]. PharmacoEconomics, 2022, 40(10): 957-969.
- Mao Z, Ahmed S, Graham C, et al. The unfolding method to explore health-related quality of life constructs in a Chinese general population[J]. Value in Health, 2021, 24(6): 846-854
- Mao, Z., Ahmed, S., Graham, C., & Kind, P. (2020). Exploring subjective constructions of health in China: a Q-methodological investigation. Health Qual Life Outcomes, 18(1), 165.[11]
- Development and Evaluation of a General Quality of Life Scale for Chinese People. Zhong hua liu xing bing xue za zhi(10), 27-32.[23]

**3 stress**

- Mao Z, Ahmed S, Graham C, et al. The unfolding method to explore health-related quality of life constructs in a Chinese general population[J]. Value in Health, 2021, 24(6): 846-854
- Wu Y., Xie G., Li Y, Zhou B., Zhang P., Shi P, Ren F., Ma L.(2005). Development and Evaluation of a General Quality of Life Scale for Chinese People. Zhong hua liu xing bing xue za zhi(10), 27-32.[23]
- Mao, Z., Ahmed, S., Graham, C., & Kind, P. (2020). Exploring subjective constructions of health in China: a Q-methodological investigation. Health Qual Life Outcomes, 18(1), 165.[11]

**2 anxiety**

- Mao Z, Ahmed S, Graham C, et al. The unfolding method to explore health-related quality of life constructs in a Chinese general population[J]. Value in Health, 2021, 24(6): 846-854
- Mao, Z., Ahmed, S., Graham, C., & Kind, P. (2020). Exploring subjective constructions of health in China: a Q-methodological investigation. Health Qual Life Outcomes, 18(1), 165.[11]

**2 regularity in daily life**

- Mao Z, Ahmed S, Graham C, et al. The unfolding method to explore health-related quality of life constructs in a Chinese general population[J]. Value in Health, 2021, 24(6): 846-854
- Mao, Z., Ahmed, S., Graham, C., & Kind, P. (2020). Exploring subjective constructions of health in China: a Q-methodological investigation. Health Qual Life Outcomes, 18(1), 165.[11]

**2 sex life**

- Mao Z, Ahmed S, Graham C, et al. The unfolding method to explore health-related quality of life constructs in a Chinese general population[J]. Value in Health, 2021, 24(6): 846-854
- Wu Y., Xie G., Li Y, Zhou B., Zhang P., Shi P, Ren F., Ma L.(2005). Development and Evaluation of a General Quality of Life Scale for Chinese People. Zhong hua liu xing bing xue za zhi(10), 27-32.[23]

**2 loneliness**

- Mao Z, Ahmed S, Graham C, et al. The unfolding method to explore health-related quality of life constructs in a Chinese general population[J]. Value in Health, 2021, 24(6): 846-854
- Wu Y., Xie G., Li Y, Zhou B., Zhang P., Shi P, Ren F., Ma L.(2005). Development and Evaluation of a General Quality of Life Scale for Chinese People. Zhong hua liu xing bing xue za zhi(10), 27-32.[23]

**1 worry**

- Wu J, He X, Chen P, et al. China Health Related Outcomes Measures (CHROME): Development of a New Generic Preference-Based Measure for the Chinese Population[J]. PharmacoEconomics, 2022, 40(10): 957-969.

**1 anger**

- Mao Z, Ahmed S, Graham C, et al. The unfolding method to explore health-related quality of life constructs in a Chinese general population[J]. Value in Health, 2021, 24(6): 846-854

**1 fear**

- Mao Z, Ahmed S, Graham C, et al. The unfolding method to explore health-related quality of life constructs in a Chinese general population[J]. Value in Health, 2021, 24(6): 846-854

**1 happy mood**

- Li, M., Bao, Z., Lv, G., Zhou, J., Chen, P., & Luo, N. (2021). Culture-Related Health Disparities in Quality of Life: Assessment of Instrument Dimensions Among Chinese. Front Public Health, 9, 663904.[10]

**1 no worry**

- Li, M., Bao, Z., Lv, G., Zhou, J., Chen, P., & Luo, N. (2021). Culture-Related Health Disparities in Quality of Life: Assessment of Instrument Dimensions Among Chinese. Front Public Health, 9, 663904.[10]

**1 no pressure**

- Li, M., Bao, Z., Lv, G., Zhou, J., Chen, P., & Luo, N. (2021). Culture-Related Health Disparities in Quality of Life: Assessment of Instrument Dimensions Among Chinese. Front Public Health, 9, 663904.[10]

**3 self-confidence**

- Mao Z, Ahmed S, Graham C, et al. The unfolding method to explore health-related quality of life constructs in a Chinese general population[J]. Value in Health, 2021, 24(6): 846-854
- Wu Y., Xie G., Li Y, Zhou B., Zhang P., Shi P, Ren F., Ma L.(2005). Development and Evaluation of a General Quality of Life Scale for Chinese People. Zhong hua liu xing bing xue za zhi(10), 27-32.[23]
- Mao, Z., Ahmed, S., Graham, C., & Kind, P. (2020). Exploring subjective constructions of health in China: a Q-methodological investigation. Health Qual Life Outcomes, 18(1), 165.[11]

**2 morality**

- Mao Z, Ahmed S, Graham C, et al. The unfolding method to explore health-related quality of life constructs in a Chinese general population[J]. Value in Health, 2021, 24(6): 846-854
- Mao, Z., Ahmed, S., Graham, C., & Kind, P. (2020). Exploring subjective constructions of health in China: a Q-methodological investigation. Health Qual Life Outcomes, 18(1), 165.[11]

**3 positive attitude**

- Mao Z, Ahmed S, Graham C, et al. The unfolding method to explore health-related quality of life constructs in a Chinese general population[J]. Value in Health, 2021, 24(6): 846-854
- Li, M., Bao, Z., Lv, G., Zhou, J., Chen, P., & Luo, N. (2021). Culture-Related Health Disparities in Quality of Life: Assessment of Instrument Dimensions Among Chinese. Front Public Health, 9, 663904.[10]
- Mao, Z., Ahmed, S., Graham, C., & Kind, P. (2020). Exploring subjective constructions of health in China: a Q-methodological investigation. Health Qual Life Outcomes, 18(1), 165.[11]

**2 peace**

- Mao Z, Ahmed S, Graham C, et al. The unfolding method to explore health-related quality of life constructs in a Chinese general population[J]. Value in Health, 2021, 24(6): 846-854
- Mao, Z., Ahmed, S., Graham, C., & Kind, P. (2020). Exploring subjective constructions of health in China: a Q-methodological investigation. Health Qual Life Outcomes, 18(1), 165.[11]

**2 breadth of mind**

- Mao Z, Ahmed S, Graham C, et al. The unfolding method to explore health-related quality of life constructs in a Chinese general population[J]. Value in Health, 2021, 24(6): 846-854
- Mao, Z., Ahmed, S., Graham, C., & Kind, P. (2020). Exploring subjective constructions of health in China: a Q-methodological investigation. Health Qual Life Outcomes, 18(1), 165.[11]

**1sense of satisfaction of life**

- Mao Z, Ahmed S, Graham C, et al. The unfolding method to explore health-related quality of life constructs in a Chinese general population[J]. Value in Health, 2021, 24(6): 846-854

**4 ability to memory**

- Wu J, He X, Chen P, et al. China Health Related Outcomes Measures (CHROME): Development of a New Generic Preference-Based Measure for the Chinese Population[J]. PharmacoEconomics, 2022, 40(10): 957-969.
- Mao Z, Ahmed S, Graham C, et al. The unfolding method to explore health-related quality of life constructs in a Chinese general population[J]. Value in Health, 2021, 24(6): 846-854
- Wu Y., Xie G., Li Y, Zhou B., Zhang P., Shi P, Ren F., Ma L.(2005). Development and Evaluation of a General Quality of Life Scale for Chinese People. Zhong hua liu xing bing xue za zhi(10), 27-32.[23]
- Mao, Z., Ahmed, S., Graham, C., & Kind, P. (2020). Exploring subjective constructions of health in China: a Q-methodological investigation. Health Qual Life Outcomes, 18(1), 165.[11]

**3 ability to concentration**

- Mao Z, Ahmed S, Graham C, et al. The unfolding method to explore health-related quality of life constructs in a Chinese general population[J]. Value in Health, 2021, 24(6): 846-854
- Wu Y., Xie G., Li Y, Zhou B., Zhang P., Shi P, Ren F., Ma L.(2005). Development and Evaluation of a General Quality of Life Scale for Chinese People. Zhong hua liu xing bing xue za zhi(10), 27-32.[23]
- Mao, Z., Ahmed, S., Graham, C., & Kind, P. (2020). Exploring subjective constructions of health in China: a Q-methodological investigation. Health Qual Life Outcomes, 18(1), 165.[11]

**2 sharp mind**

- Mao Z, Ahmed S, Graham C, et al. The unfolding method to explore health-related quality of life constructs in a Chinese general population[J]. Value in Health, 2021, 24(6): 846-854
- Li, M., Bao, Z., Lv, G., Zhou, J., Chen, P., & Luo, N. (2021). Culture-Related Health Disparities in Quality of Life: Assessment of Instrument Dimensions Among Chinese. Front Public Health, 9, 663904.[10]

**2clear mind**

- Li, M., Bao, Z., Lv, G., Zhou, J., Chen, P., & Luo, N. (2021). Culture-Related Health Disparities in Quality of Life: Assessment of Instrument Dimensions Among Chinese. Front Public Health, 9, 663904.[10]
- Mao, Z., Ahmed, S., Graham, C., & Kind, P. (2020). Exploring subjective constructions of health in China: a Q-methodological investigation. Health Qual Life Outcomes, 18(1), 165.[11]

**2 ability to make decisions**

- Mao Z, Ahmed S, Graham C, et al. The unfolding method to explore health-related quality of life constructs in a Chinese general population[J]. Value in Health, 2021, 24(6): 846-854
- Mao, Z., Ahmed, S., Graham, C., & Kind, P. (2020). Exploring subjective constructions of health in China: a Q-methodological investigation. Health Qual Life Outcomes, 18(1), 165.[11]

**1 ability to think**

- Mao Z, Ahmed S, Graham C, et al. The unfolding method to explore health-related quality of life constructs in a Chinese general population[J]. Value in Health, 2021, 24(6): 846-854

**5social interactions**

- Shiroiwa T, Murata T, Ahn J, et al. Developing a New Region-Specific Preference-Based Measure in East and Southeast Asia[J]. Value in health regional issues, 2022, 32: 62-69
- Wu J, He X, Chen P, et al. China Health Related Outcomes Measures (CHROME): Development of a New Generic Preference-Based Measure for the Chinese Population[J]. PharmacoEconomics, 2022, 40(10): 957-969.
- Mao, Z., Ahmed, S., Graham, C., & Kind, P. (2020). Exploring subjective constructions of health in China: a Q-methodological investigation. Health Qual Life Outcomes, 18(1), 165.[11]
- Development and Evaluation of a General Quality of Life Scale for Chinese People. Zhong hua liu xing bing xue za zhi(10), 27-32.[23]
- Li, M., Bao, Z., Lv, G., Zhou, J., Chen, P., & Luo, N. (2021). Culture-Related Health Disparities in Quality of Life: Assessment of Instrument Dimensions Among Chinese. Front Public Health, 9, 663904.[10]

**3 good relationships and communications**

- Mao Z, Ahmed S, Graham C, et al. The unfolding method to explore health-related quality of life constructs in a Chinese general population[J]. Value in Health, 2021, 24(6): 846-854
- Li, M., Bao, Z., Lv, G., Zhou, J., Chen, P., & Luo, N. (2021). Culture-Related Health Disparities in Quality of Life: Assessment of Instrument Dimensions Among Chinese. Front Public Health, 9, 663904.[10]
- Mao, Z., Ahmed, S., Graham, C., & Kind, P. (2020). Exploring subjective constructions of health in China: a Q-methodological investigation. Health Qual Life Outcomes, 18(1), 165.[11]

**2 social support**

- Mao Z, Ahmed S, Graham C, et al. The unfolding method to explore health-related quality of life constructs in a Chinese general population[J]. Value in Health, 2021, 24(6): 846-854
- Wu Y., Xie G., Li Y, Zhou B., Zhang P., Shi P, Ren F., Ma L.(2005). Development and Evaluation of a General Quality of Life Scale for Chinese People. Zhong hua liu xing bing xue za zhi(10), 27-32.[23]

**1 adaptability to social environment**

- Mao Z, Ahmed S, Graham C, et al. The unfolding method to explore health-related quality of life constructs in a Chinese general population[J]. Value in Health, 2021, 24(6): 846-854

**1 burdens to others**

- Shiroiwa T, Murata T, Ahn J, et al. Developing a New Region-Specific Preference-Based Measure in East and Southeast Asia[J]. Value in health regional issues, 2022, 32: 62-69

**2 ability to complete work and study**

- Shiroiwa T, Murata T, Ahn J, et al. Developing a New Region-Specific Preference-Based Measure in East and Southeast Asia[J]. Value in health regional issues, 2022, 32: 62-69
- Li, M., Bao, Z., Lv, G., Zhou, J., Chen, P., & Luo, N. (2021). Culture-Related Health Disparities in Quality of Life: Assessment of Instrument Dimensions Among Chinese. Front Public Health, 9, 663904.[10]

**1 economic status**

- Wu Y., Xie G., Li Y, Zhou B., Zhang P., Shi P, Ren F., Ma L.(2005). Development and Evaluation of a General Quality of Life Scale for Chinese People. Zhong hua liu xing bing xue za zhi(10), 27-32.[23]

**1 dwelling conditions**

- Wu Y., Xie G., Li Y, Zhou B., Zhang P., Shi P, Ren F., Ma L.(2005). Development and Evaluation of a General Quality of Life Scale for Chinese People. Zhong hua liu xing bing xue za zhi(10), 27-32.[23]

**1 adaptability to weather changes**

- Mao Z, Ahmed S, Graham C, et al. The unfolding method to explore health-related quality of life constructs in a Chinese general population[J]. Value in Health, 2021, 24(6): 846-854
